# Human dyskerin binds to cytoplasmic H/ACA-box-containing transcripts affecting nuclear hormone receptor dependence

**DOI:** 10.1186/s13059-022-02746-3

**Published:** 2022-08-22

**Authors:** Federico Zacchini, Giulia Venturi, Veronica De Sanctis, Roberto Bertorelli, Claudio Ceccarelli, Donatella Santini, Mario Taffurelli, Marianna Penzo, Davide Treré, Alberto Inga, Erik Dassi, Lorenzo Montanaro

**Affiliations:** 1 Dipartimento di Medicina Specialistica, Diagnostica e Sperimentale (DIMES), Alma Mater Studiorum - Università di Bologna, I-40138 Bologna, Italy; 2 Centro di Ricerca Biomedica Applicata – CRBA, Università̀ di Bologna, Policlinico di Sant’Orsola, I-40138 Bologna, Italy; 3 Dipartimento di Biologia Cellulare, Computazionale e Integrata (CIBIO), Università di Trento, I-38123 Trento, Italy; 4 Unità Operativa di Anatomia e Istologia Patologica, IRCCS Azienda Ospedaliero-Universitaria di Bologna, Via Albertoni 15, I-40138 Bologna, Italy; 5 Unità Operativa di Chirurgia Senologica, IRCCS Azienda Ospedaliero-Universitaria di Bologna, Via Albertoni 15, I-40138 Bologna, Italy; 6 Dipartimento di Scienze Mediche e Chirurgiche (DIMEC), Alma Mater Studiorum - Università di Bologna, I-40138 Bologna, Italy; 7 Departmental Program in Laboratory Medicine, IRCCS Azienda Ospedaliero-Universitaria di Bologna, Via Albertoni 15, I-40138 Bologna, Italy

**Keywords:** DKC1, intron retention, RNA binding, Post-transcriptional control, Breast cancer

## Abstract

**Background:**

Dyskerin is a nuclear protein involved in H/ACA box snoRNA-guided uridine modification of RNA. In humans, its defective function is associated with cancer development and induces specific post-transcriptional alterations of gene expression. In this study, we seek to unbiasedly identify mRNAs regulated by dyskerin in human breast cancer-derived cells.

**Results:**

We find that dyskerin depletion affects the expression and the association with polysomes of selected mRNA isoforms characterized by the retention of H/ACA box snoRNA-containing introns. These snoRNA retaining transcripts (snoRTs) are bound by dyskerin in the cytoplasm in the form of shorter 3′ snoRT fragments. We then characterize the whole cytoplasmic dyskerin RNA interactome and find both H/ACA box snoRTs and protein-coding transcripts which may be targeted by the snoRTs’ guide properties. Since a fraction of these protein-coding transcripts is involved in the nuclear hormone receptor binding, we test to see if this specific activity is affected by dyskerin. Obtained results indicate that dyskerin dysregulation may alter the dependence on nuclear hormone receptor ligands in breast cancer cells. These results are paralleled by consistent observations on the outcome of primary breast cancer patients stratified according to their tumor hormonal status. Accordingly, experiments in nude mice show that the reduction of dyskerin levels in estrogen-dependent cells favors xenograft development in the absence of estrogen supplementation.

**Conclusions:**

Our work suggests a cytoplasmic function for dyskerin which could affect mRNA post-transcriptional networks relevant for nuclear hormone receptor functions.

**Supplementary Information:**

The online version contains supplementary material available at 10.1186/s13059-022-02746-3.

## Background

Dyskerin is a conserved, multifunctional protein encoded by the DKC1 gene [1]. DKC1 mutations cause the rare multisystemic syndrome X-linked dyskeratosis congenita [1]. In addition, dyskerin expression is often dysregulated in human cancer [2].

Major dyskerin functions include telomerase complex stabilization [3] and the site-specific isomerization of uridine to pseudouridine in RNA molecules [4]. These functions are achieved by dyskerin binding to a class of noncoding small nucleolar RNAs termed H/ACA box snoRNAs, which also include the telomerase RNA component (TERC). Dyskerin binds these RNA molecules in association with other pseudouridylation complex core proteins, namely NHP2, NOP10, and GAR1 [5, 6].

On the basis of their specific sequence, most H/ACA box snoRNAs guide the pseudouridylation complex on specific uridine residues for their modification to pseudouridine. The majority of these target uridines lie on ribosomal RNA (rRNA) [7].

In vertebrates, most of H/ACA box snoRNA genes are present as intronic sequences contained in a subset of essential genes involved in the synthesis or functioning of the translational apparatus, including those coding for ribosomal proteins, translation factors, nucleolar proteins, and proteins involved in mRNA binding, transport, and stability. H/ACA box snoRNAs are then transcribed by RNA Pol II and generated mainly through the splicing of the nascent pre-mRNA and the exonucleolytic trimming of the spliced intron [8–10].

Pathogenic DKC1 mutations and dyskerin depletion induce both TERC destabilization with a loss of telomerase activity [3] and a defective rRNA pseudouridylation [2, 11–13], associated with changes in mRNA translation which end up with specific alterations in gene expression. Such changes involve altered cap-independent translation initiation and reduced translational fidelity [14–18]. Notably, these effects were involved to explain the role played by dyskerin in the development of different tumor types, including breast cancer [11, 16, 17, 19].

In this study, we sought to identify mRNAs affected by dyskerin depletion at the transcriptome-wide level. We thus performed an RNA-seq analysis of total and polysome-associated RNAs in control and stably dyskerin-depleted breast cancer-derived cells. Results showed that dyskerin downregulation strongly affects the recruitment to polysomal fractions of mRNA isoforms characterized by the retention of H/ACA box snoRNA sequences containing introns. Further analyses indicated that dyskerin binds to these snoRNA retaining transcripts (snoRTs) in the cytoplasm and is involved in the regulation of a complex RNA interactome affecting mRNA post-transcriptional networks, which are important for nuclear hormone receptor functions.

## Results

### Dyskerin regulates the recruitment to polysomes of snoRTs

**Fig. 1:**
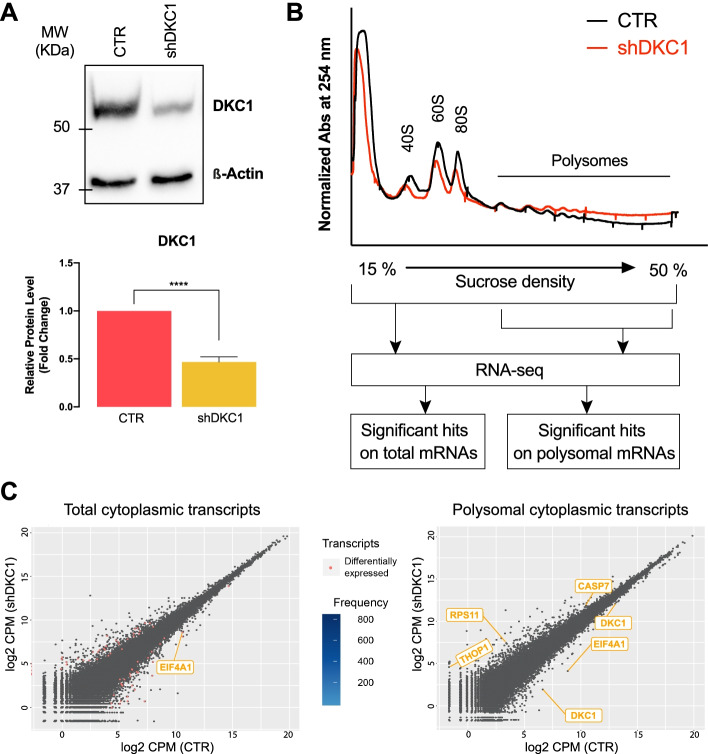
Dyskerin regulates the recruitment to polysomes of snoRTs. **A** Representative Western blot analysis image (top) and densitometric analysis of 5 independent replicates (bottom) of dyskerin levels after DKC1 mRNA KD in MCF7 cells. Data are shown as means + SD. Paired Student’s *t* tests were performed relative to controls. ****p < 0.001. **B** Representative polysome profiles obtained by 15–50% sucrose density gradient centrifugation from control (black) and DKC1 KD (red) MCF7 cells. Three independent replicates were used. Ten percent of each fraction was pooled to reconstitute total mRNA, while the remaining polysomal fractions were pooled together. Reverse transcribed polysomal and total mRNAs were sequenced using a next-generation sequencing (RNA-seq) approach with a depth of about 50–60 M usable reads. Processed data were analyzed for significant differences in mRNA levels normalized signals between polysomal and total RNA fractions. **C** Count-based differential expression analysis of total cytoplasmic (left panel) and polysomal-recruited (right panel) transcripts. Differentially expressed transcripts are depicted as red dots, while transcripts gene names of interest are indicated

We stably reduced the levels of dyskerin in breast carcinoma-derived MCF7 cells by specific shRNA expression as previously performed [17] (Fig. 1A). These cells, and their relevant controls, were used to isolate total and actively translated cytoplasmic mRNAs by polysomal fractionation. PolyA - RNA-Seq was then performed for an unbiased identification of mRNAs whose translation was affected by dyskerin depletion (Fig. 1B). First, the results were analyzed at the gene level, showing no significant difference in total and polysomal fractions (except for DKC1) after false discovery rate correction (Additional file 1: Fig. S1A). Then, the differences involving specific mRNA splicing isoforms were investigated by both isoform-based and exon-based analyses. These analyses led to the identification of a subset of mRNA isoforms and exon sequences which are either differentially represented in total fractions or differentially recruited to polysomes after dyskerin depletion, not leading to a different expression of the protein encoded by these genes (see Fig. 1C and Additional file 1: Fig. S1B, S1C, S1D).

Interestingly, the list included several mRNA isoforms which retain introns containing H/ACA box snoRNA sequences that we defined as H/ACA snoRTs (Additional file 1: Fig. S1B, S1C, highlighted lines). In particular, we identified a noncoding isoform of the EIF4A1 mRNA which retains the intron-containing SNORA67 sequence (EIF4A1 snoRT), a coding isoform of RPL32 mRNA retaining SNORA7A (RPL32 snoRT) in its 5′ UTR, and noncoding isoforms of TAF1D and DKC1 mRNAs retaining several H/ACA box snoRNAs (see Additional file 1: Fig. S1B, S1C for details). The effect of dyskerin depletion on the polysomal recruitment of these snoRTs was further validated by RT-qPCR using different siRNA sequences to reduce DKC1 levels in MCF7 and also MDA-MB-231 breast cancer cells (Additional file 1: Fig. S1E).

Given dyskerin’s ability to bind to H/ACA box snoRNAs sequences in the nucleus, our results suggest that H/ACA snoRTs might also be bound by dyskerin.

### H/ACA snoRTs are bound by dyskerin in the cytoplasm

**Fig. 2:**
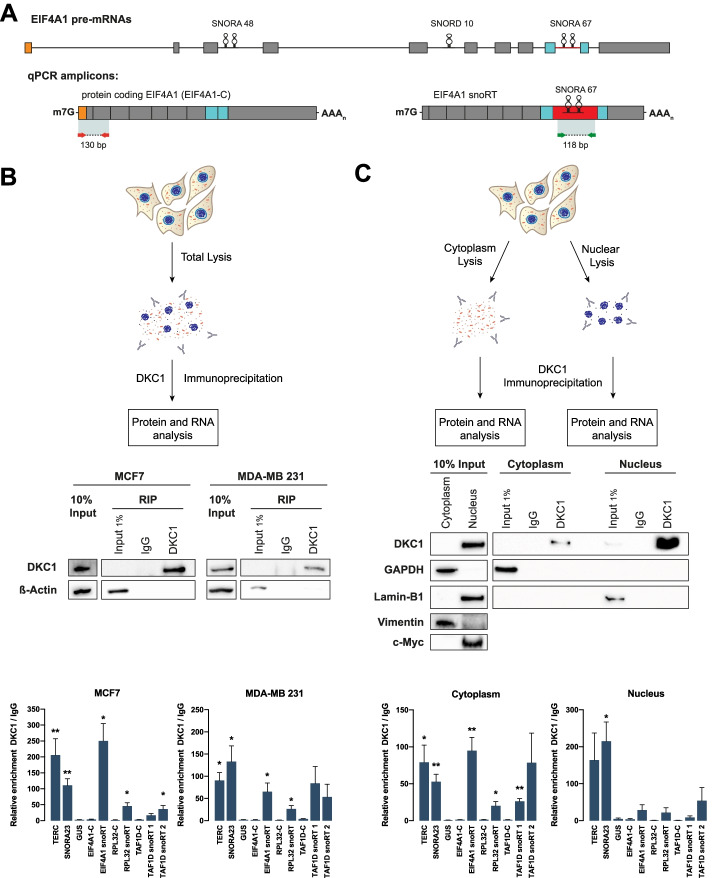
H/ACA snoRTs are bound by dyskerin in the cytoplasm. **A** In scale schematic overview of the EIF4A1 pre-mRNA. Introns are depicted as lines connected to exons. SNORA48, SNORD10, and SNORA67 are shown. Intron-containing-SNORA67 sequence is depicted as a red line in pre-mRNA or as a red box in the EIF4A1 snoRT intron-retaining transcript. Exons flanking intron-containing-SNORA67 sequence are depicted as blue boxes. The first exon of pre-mRNA is depicted as an orange box. Diagnostic RT-qPCR amplicons are represented. The amplicon between red arrows identifies the protein coding mRNA, while the amplicon between green arrows identifies every EIF4A1 snoRTs. Primer sequences are listed in Additional file 3: Table S2. m7G: cap; AAAn: poly(A) tail; P: monophosphate. **B** RNA immunoprecipitation analysis of dyskerin from total cellular lysates. Top: outline of sample preparation steps. Middle: Western blot analysis of immunoprecipitated fractions from total MCF7 and MB-MDA 231 cell lysates using control IgG or anti-dyskerin antibody. A 10% input extract was used to verify the correct lysis. Bottom: RT-qPCR analysis of the known dyskerin targets (TERC, SNORA23), a known off-target (GUS), and transcripts of interest on MCF7 (left) and MDA-MB 231 (right) cell lines. Results are expressed as the fold change of dyskerin immunoprecipitation RNA level against immunoprecipitation with IgG. Data are shown as means + SEM. *n*=4 biological replicates were performed for each experiment. Unpaired Student’s *t* tests were performed on respective controls IgG. **C** RNA immunoprecipitation analysis of dyskerin from cytoplasmic and nuclear cell lysates. Top: outline of sample preparation steps. Centre: Western blot analysis of immunoprecipitated fractions from cytoplasmic and nuclear MCF7 cell lysates using control IgG or anti-dyskerin antibody. GAPDH was used as cytoplasmic marker, while Lamin-B1 was used as nuclear marker. A 10% input extract was used to verify the correct lysis. Bottom: RT-qPCR analysis of the known dyskerin targets (TERC, SNORA23), a known off-target (GUS), and transcripts of interest of cytoplasmic (left) and nuclear (right) RIP analysis. Results are expressed as the fold change of dyskerin immunoprecipitation RNA level against immunoprecipitation with IgG. Data are shown as means + SEM. *n*=4 biological replicates were performed for each experiment. Unpaired Student’s *t* tests were performed on respective controls IgG. EIF4A1-C *p < 0.05, **p < 0.01, ***p < 0.005, ****p < 0.001

To investigate the association of dyskerin with H/ACA snoRTs, we performed an RNA immunoprecipitation (RIP) using an anti-dyskerin antibody followed by RT-qPCR. For this purpose, we used primers capable of distinguishing the canonical (EIF4A1-C, RPL32-C, TAF1D-C) and intron-retaining isoforms (EIF4A1-snoRT, RPL32-snoRT, TAF1D-snoRT 1, TAF1D-snoRT 2) of interest (Fig. 2A, Additional file 1: Fig. S2A-B). The results obtained on MCF7 and MDA-MB-231 breast cancer-derived cells indicate that H/ACA snoRTs are indeed associated to dyskerin. This resembles what occurs with other known H/ACA box snoRNAs, such as TERC and SNORA23 (notably, the independently transcribed TERC sequence is not reported as being part of any known cellular mRNA—Fig. 2B). In principle, the qPCR primers we used to detect the snoRTs could also amplify the unspliced pre-mRNA. To clarify this point, we looked in the dyskerin IP for the presence of other introns not containing any snoRNA sequence that could derive from the genes transcribing for the identified snoRTs (EIF4A1, RPL32, and TAF1D). Obtained results indicated that dyskerin is not associated to these pre-mRNAs (Additional file 1: Fig. S2C). In addition, since we detected the H/ACA snoRTs in polysomal fractions that are localized in the cytoplasm we investigated if their interaction with dyskerin takes place also in this cellular compartment. A second RIP analysis was then performed after subcellular fractioning. To ensure proper fractioning and limited leakage of nuclear content in the cytoplasm we searched for both anchored (e.g., vimentin, pEGFR in the cytoplasm, and lamin-B1 in the nucleus) and soluble (e.g., GAPDH in the cytoplasm, c-Myc, c-Jun, and PARP1 in the nucleus) proteins as controls. The quality of the fractioning was also confirmed evaluating RNA species known to localize prevalently either in the cytoplasm (ACTB and coding EIF4A1 mRNAs) or in the nucleus (MALAT1 and SNORA23 RNA) (Additional file 1: Fig. S2D). Our results showed that the association of H/ACA snoRTs to dyskerin occurs also in the cytoplasm and that for some of these transcripts (such as EIF4A1 snoRT) this interaction is detected preferentially in this cellular compartment (Fig. 2C, Additional file 1: Fig. S2E). Worthy of note, despite its known nuclear localization, we identified detectable amounts of the 57 kDa full-length dyskerin protein by Western blot analysis after DKC1 immunoprecipitation performed in cytoplasmic fractions (Fig. 2C). The presence of dyskerin in the cytoplasm was also independently confirmed by immunofluorescence microscopy (Additional file 1: Fig. S2F).

We also performed RIP analyses by using antibodies directed to the other pseudouridylation complex core proteins NHP2 and GAR1. Results showed that EIF4A1 snoRT was associated to these proteins as well, indicating that H/ACA snoRTs are bound by pseudouridylation core proteins (Additional file 1: Fig. S2G).

### The dyskerin-bound EIF4A1 snoRT is truncated at its 5′ end

**Fig. 3:**
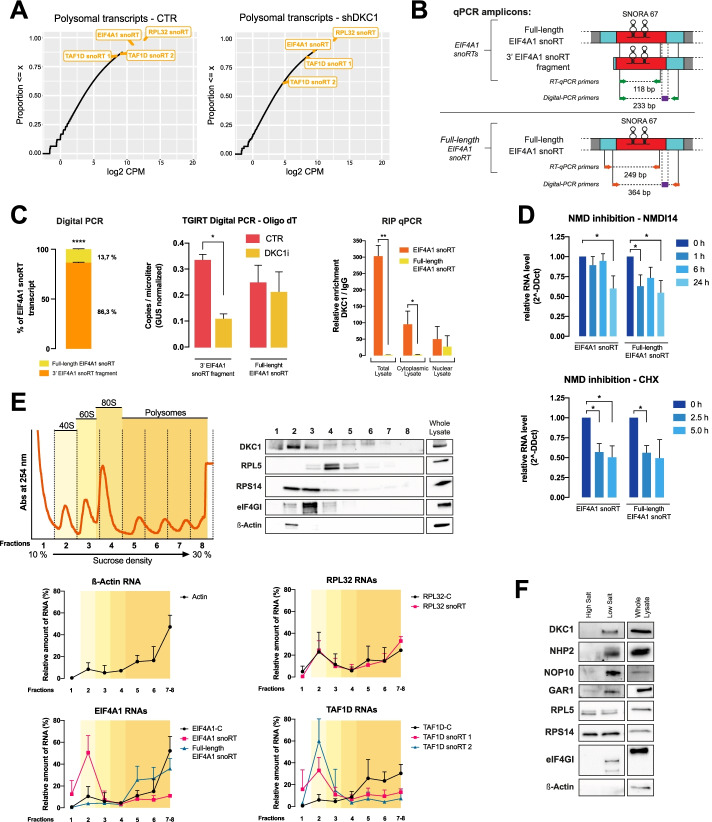
The dyskerin-bound EIF4A1 snoRT is truncated at its 5′ end and interacts with ribosomes in the cytoplasm. **A** Empirical cumulative distribution function of total cytoplasmic and polysomal-recruited transcripts. Transcripts of interest are highlighted in boxes. **B** RT-qPCR amplicons and relative mRNA species identified by designed primers. Exons flanking intron-containing-SNORA67 sequence are depicted as blue boxes, while the retained intron is depicted as a red box. The amplicon between green arrows identifies every EIF4A1 snoRTs (full-length EIF4A1 snoRT and 3′ EIF4A1 snoRT fragment), while the amplicon between orange arrows identifies only the full-length EIF4A1 snoRT. In purple are depicted the probes designed for digital-PCR. Primer and probe sequences are listed in Additional file 3: Table S2. **C** Left: Percentage representation of EIF4A1 snoRTs obtained by digital PCR absolute quantification in MCF7 cells. Data are shown as the percentage of EIF4A1 snoRT after normalization on GUS housekeeping transcript. Middle: digital PCR absolute quantification of cDNA obtained by TGIRT reverse transcriptase using oligo dT in MCF7 cells after siRNA silencing of DKC1. Data are shown as copies/microliter after normalization on GUS housekeeping transcript Right: RT-qPCR analysis of EIF4A1 snoRTs performed on samples obtained by RNA immunoprecipitation of dyskerin from MCF7 total cellular lysate, cytoplasmic and nuclear fractioning. The means from three biological replicates (*n* = 3) are shown; error bars represent SD. Paired Student’s t tests were performed relative to controls. *p < 0.05, **p < 0.01, ***p < 0.005, ****p < 0.001. **D** RT-qPCR of total RNA from MCF7 cells treated with NMDI specific inhibitors NMDI14 at 25 μM for 0, 1, 6, and 24 h (up) or with translation inhibitors cycloheximide (CHX) at 25 μg/ml for 0, 2.5, or 5 h (down). The means from three biological replicates (*n* = 3) are shown; error bars represent SD. Paired Student’s t tests were performed in respect of the 0 h time point. *p < 0.05, **p < 0.01, ***p < 0.005, ****p < 0.001. **E** Polysome profiling analysis. Top left: representative polysome profile from MCF7 cells obtained by 10–30% sucrose density gradient centrifugation. The portions of the profile referring to different ribosomal subunits are highlighted. Top right: distribution of dyskerin and control proteins across the gradient analyzed by Western blotting with specific antibodies. Bottom: distribution of transcripts of interest after RNA purification from gradient fractions obtained by RT-qPCR. Results are expressed as fraction (%) of the total amount of the transcript contained in the lysate. Data are shown as means ± SEM of three different biological replicates (*N* = 3). **F** Ribosome purification: Western blotting analysis of purified ribosomes from MCF7 cells shows a co-purification of all pseudouridine-RNP complex (DKC1, NHP2, NOP10, GAR1). RPL5 and RPS14 are shown as a positive control for ribosomes purification, while eIF4G is used as a control for ribosome-interacting factors.

The MCF7 RNA-Seq data reported in Fig. 1C indicate that EIF4A1 snoRT is the most regulated transcript among the identified H/ACA snoRTs after dyskerin depletion. Therefore, considering its relative abundancy (Fig. 3A), its predominantly cytoplasmic localization (Additional file 1: Fig. S2D), and the strength of its enrichment in the dyskerin immunoprecipitation fraction (Fig. 2B), we focused on this particular transcript to study in further detail the features of dyskerin-bound snoRTs.

Previous studies reported that snoRTs can be processed by non-sense-mediated decay (NMD) [20]. In some cases, as for the C/D box Nop56 snoRT, this may lead to the generation of shorter **c**ytoplasmic 5′**s**noRNA ended, 3′-**p**oly**a**denylated transcripts referred to as cSPA RNA [21, 22]. Therefore, in order to precisely characterize the sequence of the EIF4A1 snoRT associated to dyskerin, we performed a 5′-RACE analysis. This assay was performed on RNA obtained after dyskerin RIP from MCF7 cytoplasmic fractions, where EIF4A1 snoRT is particularly enriched. Obtained results clearly showed that the sequence of the EIF4A1 snoRT associated to dyskerin is truncated at the end of the EIF4A1 exon which precedes the snoRNA-containing retained intron (see Additional file 1: Fig. S3A, Fig. 3B). The primers we initially used to identify EIF4A1 snoRT did not permit distinguishing the full-length transcript from the identified truncated snoRT corresponding to a 3′ EIF4A1 fragment. Therefore, to quantify the amount of the different identified transcripts, we performed a digital RT-PCR analysis with primers capable of selectively identifying the full-length EIF4A1 snoRT and primers able to amplify all snoRTs bearing the SNORA67-containing intron (both full length and 3′ EIF4A1 snoRTs) (Fig. 3B). Results on total mRNA from MCF7 cells showed that the full-length EIF4A1 snoRT represents only 13.7% of the total EIF4A1 snoRTs. Therefore, according to the percentage difference, the 3′ EIF4A1 snoRT must account for the remaining 86.3% (Fig. 3C—left panel; similar results were observed in MDA-MB-231 cell line; Additional file 1: Fig. S3B). By a similar digital PCR approach, employing a highly processive reverse transcriptase to exclude any possible issues related to highly structured RNAs (like many snoRNAs are), we evaluated the regulation of acute siRNA mediated dyskerin depletion (see Additional file 1: Fig. S1E) on EIF4A1 derived RNA isoforms. Obtained results showed that only the 3’ EIF4A1 snoRT fragment is affected by DKC1 silencing, further demonstrating that the regulation of EIF4A1 snoRT observed with RNA-seq is actually to be ascribed to the downregulation of this specific RNA fragment (Fig. 3C—middle panel). Interestingly, we observed very similar results by performing the reverse transcription either with oligo-dTs or with random primers (see Additional file 1: Fig. S3B—right panel), adding evidence that the observed regulation actually involves mRNA isoforms and their derived fragments. Moreover, the anti-dyskerin RIP analysis of total MCF7 cellular lysate showed that while total EIF4A1 snoRT is highly enriched, most of the full-length EIF4A1 snoRT is not associated to dyskerin (Fig. 3C—right panel). Conversely, the same RIP analysis of nuclear and cytoplasmic fractions indicated that the full-length EIF4A1 snoRT is enriched only in the nuclear fraction (indicating that the full-length transcript is bound by dyskerin in the nucleus and then exported in the cytoplasm) (Fig. 3C—right panel). To assess if the binding of dyskerin to the full-length EIF4A1 snoRT in the nucleus may contribute to the retention of the snoRNA-containing intron, we assessed the levels of total and newly produced full-length EIF4A1 snoRT after DKC1 silencing. Our results show that both total and newly transcribed levels of full-length EIF4A1 snoRT transcript are not influenced by dyskerin (Additional file 1: Fig. S3C) (indicating that the intron retention is not influenced by the pre-existing levels of DKC1). In addition, the stability of the full-length EIF4A1 snoRT assessed after actinomycin D treatment was not affected by dyskerin depletion, while that of EIF4A1 snoRT (mostly represented by the 3′ EIF4A1 snoRT fragment) was found to be strongly dyskerin-dependent (Additional file 1: Fig. S3D). To preliminarily characterize the mechanisms involved in the generation of the 3′ EIF4A1 snoRT fragment we treated MCF7 cells with two different compounds able to inhibit NMD (cycloheximide [23] and NMDI14 [24]). The treatment resulted in a clear decrease of the levels of 3′ EIF4A1 snoRT fragment indicating an involvement of this pathway. This result however was not paralleled by a correspondent increase in the full-length EIF4A1 snoRT suggesting the presence of additional levels of regulation for these transcripts (Fig. 3D). Subsequently, to investigate how dyskerin regulates the association of H/ACA snoRTs to polysomes and their mutual interaction, we performed further polysomal fractionation (Fig. 3E). In this analysis, we characterized the association of dyskerin and H/ACA snoRTs with different cytoplasmic fractions by RT-qPCR. As previously observed, we found some of the EIF4A1 snoRT (i.e., accounting for both full length and 3’ truncated RNA species) associated with polysomes. However, most of the EIF4A1 snoRT co-sedimented with the free 40S ribosomal subunit. The distribution of EIF4A1 snoRT in polysomal fractions is also paralleled by dyskerin representation. Instead, the full-length EIF4A1 snoRT is mainly recruited to polysomal fractions, similarly to the canonical EIF4A1 protein-coding isoform. These observations were confirmed also for the two TAF1D snoRT isoforms (Fig. 3E). On the other hand, results for RPL32 snoRT that is expected to be coding—since the snoRNA containing intron is retained before the initiation codon—appear to behave similarly to other coding mRNAs (Fig. 3E). Furthermore, the analysis of mRNA distribution after polysomal fractionation and treatment with the translation inhibitor puromycin (Additional file 1: Fig. S3E) and the re-analysis of publicly available ribosome profiling datasets confirmed this finding (Additional file 1: Fig. S3F). Interestingly, the redistribution of EIF4A1 snoRT after puromycin treatment was again paralleled by a similar dyskerin representation. Therefore, these results indicate that the full-length snoRTs are translated while most of the 3′ snoRT fragments accumulate outside the polysomes co-sedimenting with the 40S subunit. In addition, we isolated ribosomes in low- and high-stringency purification conditions, demonstrating that all the pseudouridylation complex core proteins are associated to cytoplasmic ribosomes, paralleling what happens for other ribosome-interacting factors (e.g., eIF4GI—Fig. 3F, similar results were observed in the MDA-MB-231 cell line; Additional file 1: Fig. S3G). In addition, to evaluate the possible interplay between EIF4A1 snoRT and its host gene, and the related possible effect on cellular behavior we specifically silenced the EIF4A1 snoRT using siRNA targeting the intron. EIF4A1 snoRT downregulation induced a significant downregulation of the EIF41 protein (Additional file 1: Fig. S3H). Parallelly, a decrease in protein synthesis and a slight negative effect on cell growth were observed after EIF4A1 snoRT depletion, indicating an involvement of this non-coding isoform in regulating cellular homeostasis.

### Dyskerin binds to a complex RNA interactome in the cytoplasm

**Fig. 4:**
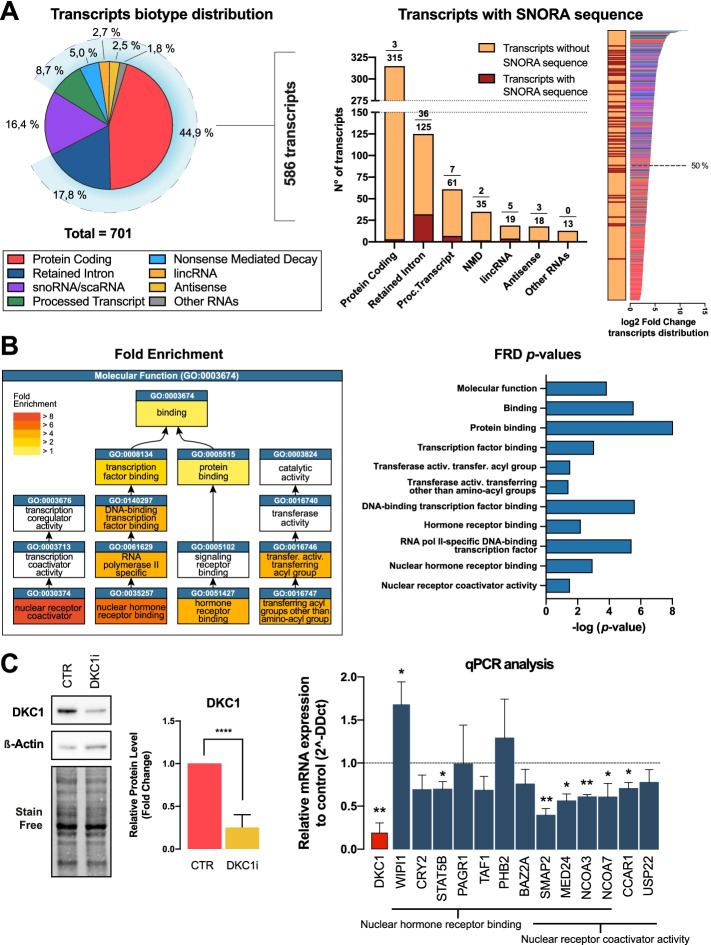
Dyskerin binds to a complex RNA interactome in the cytoplasm. **A** Left: Biotype distribution of transcripts identified by RIP-Seq analysis of RNAs co-immunoprecipitated by an anti-dyskerin antibody in a cytoplasmic MCF7 lysate. Middle: Biotype distribution of H/ACA mRNAs immunoprecipitated by dyskerin from MFC7 cytoplasmic fractions. The plot shows also transcripts with or without SNORA sequence Right: Quantitative distribution of transcripts immunoprecipitated by dyskerin from MFC7 cytoplasmic fractions. The dotted horizontal line represents the median. Five independent replicates were used for RIP-seq analysis. **B** Fold enrichment (left) and FDR *p*-values (right) of the molecular function gene ontology chart of the statistically significant terms obtained from the analysis of the 238 unique protein coding transcripts immunoprecipitated by dyskerin. FDR, False Discovery Rate. **C** Representative Western blot analysis image (left) and densitometric analysis of 5 independent replicates (right) of dyskerin levels after DKC1 mRNA RNAi in MCF7 cells. Data are shown as means + SD. Paired Student’s *t* tests were performed relative to controls. Right: RT-qPCR analysis of transcripts identified by RIP-Seq analysis belonging to the most enriched molecular functions obtained by gene ontology analysis. DKC1 mRNA silencing level is highlighted in red. Data are shown as a fold change of DKC1 RNAi MCF7 cells relative to their controls. The means from three biological replicates (*n* = 3) are displayed, error bars represent SD. Paired Student’s *t* tests were performed relative to not DKC1 interfered controls. *p < 0.05, **p < 0.01, ***p < 0.005, ****p < 0.001.

To unbiasedly characterize the transcripts bound by dyskerin in the cytoplasm in addition to the ones identified above, we performed a RIP-Seq analysis of the RNAs co-immunoprecipitated by an anti-dyskerin antibody from the cytoplasmic fraction of MCF7 cells. In this way, we identified 701 significantly enriched transcripts. The biotype distribution of the identified transcripts, according to ENSEMBL annotation [25], is shown in Fig. 4A, left panel. Of the 701 transcripts, 115 (16.4%) were univocally classified as snoRNA and scaRNA (111 of these contain an H/ACA box). Therefore, those transcripts are expected to be bound directly by dyskerin. Of the remaining 586 transcripts, 56 (8.0%) were H/ACA snoRTs transcribed from known snoRNA host genes. The biotype distribution of the known snoRTs immunoprecipitated by dyskerin is shown in Fig. 4A (middle panel) and S4A, where the “retained intron” biotype is the most represented (36 transcripts, 5.1%). Interestingly, these 56 H/ACA snoRTs were among the most enriched transcript isoforms after dyskerin IP (43/56 were above the median value of enrichment after IP, Fig. 4A, right panel). Additional 5′-RACE analysis performed on the two most abundant snoRTs identified by the RIP-seq analysis demonstrated that also these transcripts were present in the form of a 3′ snoRT fragment, indicating that the processing observed for EIF4A1 snoRT may also occur to other transcripts (Additional file 1: Fig. S4B).

To investigate the effects of dyskerin modulation on these immunoprecipitated transcripts, we once again looked at the RNA-Seq data initially obtained on control and DKC1-depleted MCF7 cells, focusing only on the 701 dyskerin-associated transcripts. Overall, these transcripts appear to be significantly regulated after dyskerin depletion compared to the rest of the transcriptome (Additional file 1: Fig. S4C).

However, many of the transcripts highly enriched by RIP-seq were protein-coding transcripts that do not contain any H/ACA snoRNA sequences. Interestingly, some of these transcripts are known targets of a pseudouridylation reaction. In fact, 114/701 of the immunoprecipitated transcripts are included in the list of 4128 pseudouridylated transcripts identified in a previous high-throughput pseudouridine-seq analysis [26]. We then considered the hg38 NCBI RefSeq transcriptome which includes 87935 different human transcripts (release 109.20201120) and performed a binomial analysis considering the expected frequency of pseudouridylated transcripts. This indicated that the frequency of known pseudouridylated RNAs was significantly higher than expected in dyskerin immunoprecipated transcripts (114/701, 16.3 % vs 4128/87,935, 4.7 %—*p*<0.00008; Additional file 1: Fig. S4D). We then performed a bioinformatic analysis of the sequences flanking these 114 known pseudouridines using snoGPS [27] to identify snoRNAs putatively guiding these modifications. The analysis identified for 55/114 of these flanking sequences a known guide H/ACA snoRNA targeting each specific position. (Additional file 1: Fig. S4E). For 47 of these 55 target sequences, the corresponding putative guide H/ACA snoRNA sequence was found in the RIP analysis (either as canonical H/ACA snoRNA or as snoRTs). These observations suggest that at least a fraction of the coding transcripts significantly enriched in the RIP analysis could be immunoprecipitated due to the presence of target sequences and their interaction with a corresponding guide H/ACA snoRNAs in the cytoplasm. In addition, we analyzed motifs in the sequences of the 701 immunoprecipitated transcripts, revealing 13 significant motifs, 6 of which were also identified by Kan et al. [28], and by a motif analysis, we performed on the ENCODE DKC1 eCLIP dataset. The analysis identified several non-H/ACA box motifs, suggesting the ability of DKC1 to bind to mRNAs through another mechanism different from the snoRNA-guided one (Additional file 1: Fig. S4F). The snoRNAs H/ACA box motif (ANANNA + ACA) was not identified by this analysis, due to its high degeneracy (ANANNA) and extreme shortness (ACA). We therefore separately searched for occurrence of these two motifs in the 125 retained-intron transcripts bound by DKC1. Out of these, 123 included both a match to the ANANNA motif and a downstream match to the ACA motif, 53 of which (42%) with a plausible distance between the ANANNA and the ACA site (10 <= distance <= 300). Globally, this suggests that DKC1 may bind different transcripts either through the H/ACA snoRNA binding sequences or through additional recognition sequences. To preliminary classify the binding mechanism of dyskerin with its interactome we analyzed the above-mentioned eCLIP dataset, specifically looking for the transcripts we found enriched in the RIP-Seq analysis. Most of the snoRT (52/56) and snoRNA/scaRNA sequences (104/115) that we identified in our RIP-Seq displayed an enrichment of the coverage indicating that they could be bound directly by dyskerin, while 71 additional transcripts show a similar behavior but do not retain any reported snoRNA sequence. A further analysis on these transcripts showed that 48/71 contain one of the consensus sequences described by Kan [28] or those identified in our study. Notably, while some motifs (particularly motif 1, 7, and 11 as reported in Additional file 1: Fig. S4F) were found consistently represented, others (5/13) were not represented at all in the eCLIP dataset, supporting the idea that only some of the motifs identified actually correspond to a real dyskerin binding. All the remaining transcripts (including the majority of the coding ones—174/312) do not show any enriched coverage in the CLIP analysis suggesting again they might be bound by dyskerin indirectly (a summary of this analysis is reported in table Additional file 2: Table S1). To obtain functional insights into this complex regulation, we performed a Gene Ontology analysis on the list of genes that transcribe for the mRNAs that we found associated to dyskerin. To avoid the possible confounding effect deriving from the cases where the association to dyskerin occurs through noncoding transcript isoforms (e.g., *EIF4A1*), we focused only on 238 genes corresponding to the 312 immunoprecipitated protein-coding transcripts (Fig. 4A). The most strongly enriched molecular functions identified by the analysis were “nuclear receptor coactivator activity” and “nuclear hormone receptor binding” (Fig. 4B, Additional file 1: Fig. S4G). In particular, for these two molecular functions, 13 genes were identified including SMAP2, NCOA7, NCOA3, USP22, MED24, and CCAR1 for the first and WIPI1, MED24, CRY2, STAT5B, PAGR1, TAF1, NCOA7, NCOA3, PHB2, BAZ2A, and SMAP2 for the second. The large majority (10/13) of these genes were reported to play a role in the regulation of estrogen and/or progesterone response in human breast cancer (NCOA7: [29]; NCOA3: [30]; USP22: [31]; MED24: [32]; CCAR1: [33]; CRY2: [34]; STAT5B: [35]; PAGR1: [36]; TAF1: [37]; PHB2: [38]). Interestingly, a significant fraction of these 13 transcripts are reported to be pseudouridylated in the RMBase database (3/13—23.1%, namely STA5B, PHB2, BAZ2A). The analysis of the sequences flanking these modification sites identified putative guide snoRNA/snoRTs that were all enriched in the above reported RIP-seq analysis (see Additional file 1: Fig. S4E), suggesting that the modification of these transcripts could be RNA dependent. In addition, transient acute dyskerin depletion after siRNA transfection was able to quantitatively modulate 7/13 of these genes, being 6 significantly downregulated and 1 significantly upregulated (Fig. 4C).

### Dyskerin modulates nuclear hormone receptor-mediated dependence in human breast cancer cells

**Fig. 5:**
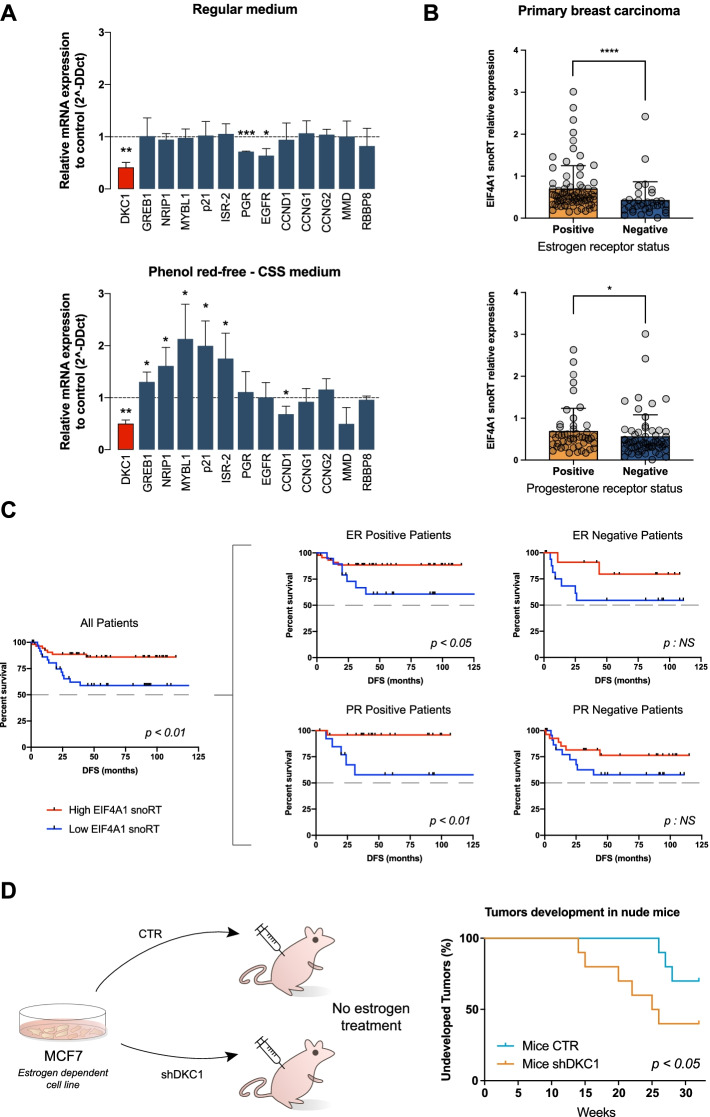
Dyskerin levels modify nuclear hormone receptor-mediated dependence in human breast cancer cells. **A** RT-qPCR analysis of selected known target transcripts of estrogen and progesterone receptors, observed under standard medium condition or with a phenol red-free and charcoal-stripped serum (CSS) medium condition. Data are shown as a fold change of shDKC1 MCF7 cells relative to their controls. The means from three biological replicates (*n* = 3) are displayed, error bars represent SD. Paired Student’s *t* tests were performed relative to not DKC1 interfered controls. **B** EIF4A1 snoRT relative expression obtained by RT-qPCR of RNA extracted from 111 breast cancer tissues. Patients are divided into positive and negative for estrogen receptors (top) or progesterone receptors (bottom). Error bars represent SD. Mann-Whitney tests were performed. **C** Kaplan-Meier survival curves for breast cancer patients. Patients are divided between high and low EIF4A1 snoRT expression (separated by the median value EIF4A1 snoRT expression) obtained by RT-qPCR analysis. The left plot shows the curves of all patients (*n* = 89) while the right plots show only the curves for estrogen receptor-positive (*n* = 62) and -negative (*n* = 27) (top), or progesterone receptor-positive (*n* = 38) and -negative (*n* = 51) (bottom) patients. Censored patients are indicated as the mark “I”. Log-rank (Mantel-Cox) test were performed. **D** Experimental plan (left). MCF7 cells (which are dependent on estrogen for their tumorigenic potential) were injected subcutaneously into mice (10 replicates for CTR and 10 replicates for shDKC1). No supplementary estrogen was administered. Kaplan-Meier curves (right) show the development of tumors in nude mice xenografted with control and shDKC1 MCF7 cells. Gehan-Breslow-Wilcoxon test were performed. *p < 0.05, **p < 0.01, ***p < 0.005, ****p < 0.001, NS (not significant)

The above-identified dyskerin interactions on nuclear hormone receptor functions guided us at investigating the effect of removing the ligands of these receptors from the serum used in in vitro experiments with shDKC1 MCF7 cells and their relevant controls. The results obtained indicate that when cultured with the addition of full serum, dyskerin depletion did not affect the expression of selected known direct targets of the two main hormone receptors active in MCF7 cells, estrogen receptor—ER—and progesterone receptor—PGR. Conversely, under charcoal treatment conditions, which remove nuclear hormone receptor ligands, at least half of the tested targets were influenced by dyskerin depletion (Fig. 5A). These results offer a preliminary indication that, in breast cancer cells, the lack of dyskerin-mediated regulation may confer some degree of estrogen and progesterone independence. To evaluate this possibility, we retrospectively analyzed a series of primary breast carcinomas available at our institution. To obtain indications on the interaction of dyskerin with the cytoplasmic mRNAs identified in the RIP-Seq analysis, we used RT-qPCR to quantify the levels of the EIF4A1 snoRT in this series. We chose the EIF4A1 snoRT as it is one of the most abundant and quantitatively regulated dyskerin-bound cytoplasmic RNAs. In this analysis, therefore, EIF4A1 snoRT levels were considered to be a proxy for dyskerin cytoplasmic RNA-binding. In the analyzed series, EIF4A1 snoRT levels significantly correlated with DKC1 mRNA levels (Additional file 1: Fig. S5A—such correlation was also confirmed on an independent 1085 cases TCGA breast invasive carcinomas dataset by GEPIA2 [39]). In addition, the results obtained showed that EIF4A1 snoRT levels were significantly and directly related to the ER and PGR labeling index obtained at the time of diagnosis by immunohistochemistry (Fig. 5B). EIF4A1 snoRT levels in tumor specimens also proved to be strongly associated to patients’ specific survival (Fig. 5C), mirroring the ER and PGR status [40]. Importantly, the prognostic value of EIF4A1 snoRT was particularly evident in ER-positive and PGR-positive cases (Fig. 5C), suggesting that the alteration in dyskerin cytoplasmic functions may be associated with estrogen and progesterone independence. In order to connect this observation to the identified dyskerin-RNA interactome, we evaluated the expression levels of a molecular signature comprising the 6 previously identified genes involved in “nuclear receptor coactivator activity” and “nuclear hormone receptor binding” molecular functions that we found associated to dyskerin and that are significantly downregulated after DKC1 depletion (i.e., STAT5B, SMAP2, MED24, NCOA3, NCOA7, and CCAR1). The analysis of the TCGA breast invasive carcinomas dataset by GEPIA2 [39] indicated that the identified molecular signature is significantly related to DKC1 expression also in this dataset (Additional file 1: Fig. S5B). In addition, we observed a strong association with the survival of patients in the sub-group of luminal B tumors, a breast cancer molecular subtype characterized by positive ER and PGR status and a relatively unfavorable prognosis (Additional file 1: Fig. S5C). This observation provides a further element in support of a possible role of the identified dyskerin cytoplasmic functions in regulating estrogen dependence in breast cancer cells. Finally, to obtain experimental in vivo evidence on this point, we xenografted nude mice subcutaneously with MCF7 shDKC1 and control cells in the absence of any additional estrogen treatment. In fact, the parental MCF7 cell line is known to be highly estrogen-dependent and to be tumorigenic in nude mice subcutaneously only upon continuous treatment with estrogen supplementation after the xenografting procedure [41]. Control cells in these conditions consistently generated tumors only in a minimal number of cases and after a long observation time (3/10 after 26 weeks). In contrast, shDKC1 cells displayed a significantly higher tumorigenic potential (6/10, *p*= 0.04) (Fig. 5D). Altogether, these results suggest that the dysregulation of the cytoplasmic RNA-binding by dyskerin alters the dependence of breast cancer cells on nuclear hormone receptor ligands.

## Discussion

With the intent of characterizing the molecular mechanisms underlying the biological and prognostic role played by dyskerin in human breast carcinomas, the present study initially aimed at identifying genes whose expression is modulated by dyskerin in breast cancer-derived cells. The unbiased analysis performed on total and polysomal RNA fractions identified a group of transcripts retaining introns with H/ACA box snoRNA sequences, that we defined as snoRTs, impacted by dyskerin depletion. However, known IRES-containing dyskerin translational targets such as Bcl-XL, XIAP, p27 [14], p53 [16], and VEGF [17] were not identified in our analysis. This might be explained by the fact that to identify yet undescribed translational targets in our study, exponentially growing cells were analyzed without inducing those stress conditions that may elicit cap-independent translation. H/ACA snoRTs were identified in the cytoplasm (and, to some extent, on polysomes), and turned out to be bound by dyskerin. In this regard, it is worth noting that, although a minimal leakage of soluble nuclear components in the cytoplasm cannot be completely ruled out based on our fractioning conditions, our controls indicate that this occurrence is extremely limited, while the identified H/ACA snoRTs are instead highly abundant. Furthermore, the presence of dyskerin in the cytoplasm was also confirmed by immunofluorescence microscopy. This finding is also in line with what has been recently reported for a sub-class of H/ACA snoRNA-bearing transcripts encoded as stable lariats that can be found in the cytoplasm associated with ectopically expressed snoRNA binding proteins such as dyskerin and GAR1 as we observed [42]. Although dyskerin is well-known to be localized in the nucleus, a previous study identified a cytoplasmic isoform of this protein characterized by a lower molecular weight [43]. In our analyses, however, the molecular weight of the dyskerin product identified in the cytoplasm corresponds to that of the canonical full-length protein. This indicates that, in addition to its nuclear localization, a fraction of cellular dyskerin is involved in mRNA binding in the cytoplasm. Our observations match what has been described for the 2′-O-methyltransferase core proteins, which are found bound to a retained intron containing a C/D snoRNA sequence in a snoRT transcribed from the NOP56 gene [22] which had been localized in the cytoplasm, as well. It has also been reported that this specific transcript is processed by NMD to a cSPA RNA. Our results, obtained on 3 abundantly represented snoRTs, are compatible with the occurrence of similar mechanisms also for H/ACA snoRTs in association with dyskerin. At this regard, our results obtained after NMD inhibition indicate that this pathway is in fact involved but cannot exclude that also other mechanisms of RNA decay such as No-Go Decay [44, 45] and/or ribosome-associated quality control pathway may occur [46]. Consistently, the EIF4A1 snoRT was found to be recruited to polysomes, a step necessary for NMD. Additional elements that should be considered regarding the similarity between the previous finding on NOP56 snoRT and our current observations include a possible self-regulatory loop involving EIF4A1 snoRT and its host gene. On the other hand, the fact that we identified a group of intron retaining transcripts associated with dyskerin while NOP56 was found associated only with its host gene represents a difference from this previously mentioned report [22].

On the basis of these previously uncovered dyskerin functions in the cytoplasm, we then comprehensively characterized the RNA molecules interacting with dyskerin in the cytoplasm via a RIP-seq analysis from cellular cytoplasmic fractions. We identified 701 significantly enriched transcripts, among which 50 H/ACA snoRTs were found. With very few exceptions, intron retention introduces a premature stop codon into the host gene transcript, thus preventing protein expression. It can be assumed, therefore, that intron retention in snoRT host genes does not directly contribute to protein diversity. On the same issue, from a more general literature analysis, it turns out that snoRTs are abundantly represented in different cell types and tissues, also from non-pathological sources, suggesting they should not be considered as a result of an infrequent splicing anomaly [47, 48] In addition, 312 protein-coding transcripts not containing any known H/ACA box snoRNA sequence were also co-immunoprecipitated. Interestingly, some of these transcripts are known targets of a pseudouridylation reaction [26] that, on the basis of the sequences flanking the modified uridines, may be guided by the snoRTs found in the RIP. Therefore, the interaction of at least a fraction of these coding mRNAs with dyskerin may be mediated by the guide sequences present in the snoRTs bound by dyskerin, suggesting for them a post-transcriptional regulatory function. In addition, it has been recently reported that dyskerin may interact directly with some mRNAs such as those encoding for a subset of ribosomal proteins including RPL10A, RPL22L1, RPL34, and RPS3 [28]. These transcripts were not enriched in our cytoplasmic RIP-seq analysis. This can be explained by the possibility that such an interaction takes place in the nucleus. It cannot be excluded, however, that a similar direct binding may underlie to the interaction of dyskerin with another subset of transcripts found in our RIP-seq analysis.

Interestingly, a significant fraction of immunoprecipitated transcripts encode for proteins involved in mediating the effect of nuclear hormone receptor ligands.

Interestingly, our results suggest that, in breast cancer cells, the lack of cytoplasmic dyskerin functions may provide a way for luminal hormone-receptor positive tumors to escape the hormone dependence, thus becoming more aggressive. Such an effect may contribute to explaining the well-known role of dyskerin in mammary tumorigenesis [2, 11, 17]. In addition, the detection of a hormone-independence mechanism may contribute to better patient stratification. Indeed, identifying estrogen receptor-positive tumors not expected to respond to antiestrogen therapy is a long-standing diagnostic problem and may greatly contribute to a correct therapeutic tailoring [49]. In this regard, we recognize that it is not always possible, when looking at patient-derived material, to dissect dyskerin cytoplasmic-specific functions from the well-established effects on telomerase activity and rRNA pseudouridylation on the basis of DKC1 expression. Nevertheless, the evaluation of the identified gene expression signature may provide more specific information in this sense.

Our results may also be of importance for X-linked dyskeratosis congenita, caused by point mutations in the DKC1 gene [1]. In the present study, we do not investigate the effects of these mutations on the described cytoplasmic functions of dyskerin. It is worth remembering, however, that one important therapeutic option for patients with dyskeratosis congenita, as well in all the so-called telomeropathies, is treatment with androgen derivatives [50]. Therefore, the investigation of androgen response in future studies, specifically in patients bearing DKC1 mutations, appears useful. In addition, a potential nuclear hormone receptor independence in patients with DKC1 mutations may also play a role in their well-known susceptibility to developing malignancies [51]. The effect of pathogenic DKC1 mutations on this aspect also requires dedicated investigations.

## Conclusion

Our findings characterize for the first time a dyskerin-dependent mRNA post-transcriptional regulation mechanism involving snoRTs. Such a mechanism occurs in the cytoplasm and may be significant for nuclear hormone receptor function, affecting the behavior of breast cancer cells. This could contribute to explain the well-known role of dyskerin in mammary tumorigenesis representing a way for breast cancer cells to escape hormone dependency. The identification of alterations in this mechanism may also lead to better patient stratification, by identifying estrogen receptor-positive tumors not expected to respond to antiestrogen therapy.

## Methods

### Cell culture

MCF7 (female, estrogen-positive invasive breast ductal carcinoma derived) cells were cultured in RPMI 1640 medium supplemented with 10% FBS, 100 U/ml penicillin, 0.1 mg/ml streptomycin, and 2 mM L-glutamine. MDA-MB 231 (female, triple-negative invasive breast ductal carcinoma-derived) cells were cultured in DMEM containing 10% FBS, 100 U/ml penicillin, 0.1 mg/ml streptomycin, and 2 mM L-glutamine. Cells were maintained at 37°C and 5% CO_2_. Cell lines were obtained from the American Type Culture Collection (ATCC) and were routinely tested for mycoplasma using Venor®GeM Classic (Minerva Biolabs). Stable cell lines were generated by infecting/transducing MCF7 cells with a retroviral vector expressing a short hairpin RNA (shRNA) targeting DKC1, as described previously [17]. The sequence of the short hairpin oligonucleotide targeting DKC1 mRNA was 5′-CCAAGGTGACTGGTTGTTTAAT-3′. As a negative control, an empty vector was used. Stable retroviral-transduced populations were selected in a standard medium supplemented with 4 μg/ml blasticidin. For siRNA-mediated depletions, cells were transfected with siRNAs targeting DKC1 (Invitrogen, catalogue number HSS102781: 5′-AACACCUGGAAGCAUAAUCUUGGCC-3′, HSS102782: 5′-UAAACAACCAGUCACCUUGGGAUCC-3′, HSS102785: 5′-GAAGUCACAACAGAGUGCAGGCAAA-3′) or EIF4A1 snoRT (custom IDT siRNA, specifically designed for targeting intronic portion of the SNORA67 intron—for sequences see Additional file 2: Table S1) and an appropriate control (Cat. N° 12935300 - Invitrogen) using Lipofectamine RNAiMAX reagent (Invitrogen) in OptiMEM medium (Invitrogen) according to the manufacturer’s instructions. Cells were harvested 72 h after the transfection. For experiments that required the removal of nuclear hormone receptors ligands, MCF7 cells with stable dyskerin depletion and the relevant control were cultured in phenol red-free medium containing 10% charcoal-treated FBS (Brand) for 4 days.

### Cells lysis and subcellular fractionation

MCF7 and MDA-MB 231 cells were lysed with different buffers: for total protein recovery (for western blot analysis) cells were lysed in RIPA buffer (50 mM Tris-HCl pH 7.5, 150 mM NaCl, 1% IGEPAL, 0.1%, and protease inhibitor cocktail) according to a standard procedure. For the RNA Immunoprecipitation assay from the whole cellular lysate, cells were washed in phosphate-buffer saline (PBS), collected by scraping, and dissolved in Immunoprecipitation Buffer (25 mM Tris-HCl pH 7.5, 150 mM KCl, 5 mM MgCl_2_, 1mM EGTA, 10% glycerol, 1.5% IGEPAL, 0.05% SDS and protease and RNAse inhibitor cocktail) for 30 min at 4°C, followed by centrifugation at 12,000 g for 20 min at 4°C. The supernatant was collected for the total RNA IP assay. For subcellular nuclear/cytoplasmic fractioning (for the RIP and polysome assays), cells were washed in phosphate-buffer saline, collected by scraping, and dissolved in Cytoplasm Lysis Buffer (15 mM Tris-HCl pH 7.5, 7.5 mM NaCl, 1.5 mM MgCl_2_, 0.3% IGEPAL, 50 mM sucrose and protease, and RNAse inhibitor cocktail) for 15 min, followed by centrifugation at 1200 g for 15 min at 4°C. The supernatant was collected as the cytoplasmic fraction and, if needed, the pellet was washed in PBS (followed by centrifugation at 1000 g for 10 min at 4°C) and then resuspended in Nucleus Lysis Buffer (50 mM Tris-HCl pH 7.5, 50 mM KCl, 300 mM NaCl, 10% glycerol, 0.1 mM EDTA, 0.5% IGEPAL, and protease and RNAse inhibitor cocktail) for nuclear lysis, and finally centrifugated at 10000 g for 3 min at 4°C. The supernatant was collected as nuclear fraction.

### Immunofluorescence microscopy

To perform immunofluorescence staining, MCF7 cells were grown on coverslips, washed with sterile PBs, and fixed for 10 min in 4% paraformaldehyde. For standard permeabilization of membranes and nuclei, cells were incubated 10 min in 1% Triton X-100 solution. For the permeabilization of the cell membrane only, cells were incubated 10 min in 0.5 % saponin solution. After blocking with a 1% BSA, the cells were incubated overnight at 4°C with mouse monoclonal anti-dyskerin antibody (1:200) (Santa Cruz Biotechnology, sc-373956) or with mouse monoclonal anti-RPS6 antibody (1:350) (Santa Cruz Biotechnology, sc-74459). Then, cells were incubated with goat anti-mouse IgG-Alexa Fluor 488 antibody (1:250) (Invitrogen, A11029) for 1 h at 37°C. Coverslips were mounted using ProLong Gold with DAPI (Invitrogen, P36941). Microscope imaging was performed using Leica DMI4000B Inverted Fluorescence microscope (Leica Microsystems) with 40× objective, acquiring the images by identical exposure times. Image analyses and manipulation were performed using Leica LAS-X software and each image was subjected to the same adjustments in brightness and contrast.

### RNA immunoprecipitation (RIP)

For the RIP assay, a total amount of lysate corresponding to 500 μg of protein content from MCF7 and MDA-MB 231 cells was pre-cleaned with protein A/G Plus-Agarose beads (Santa-Cruz, sc-2003) for 1 h at 4°C. Five to ten percent of the pre-cleaned sample was saved as input for subsequent analysis. The remainder was used in immunoprecipitation reactions with rabbit IgG (Santa Cruz, sc-2027) or rabbit polyclonal anti-dyskerin antibody (Genetex GTX 109000) and incubated overnight at 4°C. Afterwards, the anti-dyskerin interacting fraction was captured using protein A/G Plus-Agarose beads and washed several times with Wash Buffer (25 mM Tris-HCl pH 7.5, 150 mM KCl, 5 mM MgCl_2_, 1mM EGTA, 10% glycerol and protease and RNAse inhibitor cocktail). One third of the immunoprecipitated solution was saved for Western blot analysis, while the remainder was used for RNA extraction. Likewise, after saving inputs of cytoplasmic/nuclear cell lysates, the samples were entirely subjected to the same RIP analysis.

### RNA isolation and reverse transcription

Total RNA was purified using the PureZOL™ RNA Isolation Reagent (Biorad) according to the manufacturer’s guideline. cDNA was obtained reverse-transcribing 500 ng of RNA using iScript™ cDNA Synthesis Kit (Biorad) according to the manufacturer’s instructions. The RNA derived from polysome isolation, ribosome purification, and RIP purification (and respective control/input) were purified using the standard phenol:chloroform approach, briefly: proteins were digested by incubating the sample with proteinase K (final concentration of 100 μg/ml) and SDS (final concentration of 1%) at 37°C for 1 h. Afterwards, RNA was extracted with 1/4 volume of phenol:chloroform 5:1 acid equilibrated (pH 4.7) and NaCl (final concentration of 500 mM) by spinning at 16000 g for 5 min at 4°C. Then, RNA was precipitated from the aqueous phase using isopropanol. All the RNA recovered was reverse-transcribed using iScript™ cDNA Synthesis Kit (Biorad) according to the manufacturer’s guideline. To exclude any possible issues related to highly structured RNAs, highly processive reverse transcriptase TGIRT was used [52]. Briefly: 500 ng of RNA was mixed in a specific buffer (200 mM NaCl, 5 mM MgCl_2_, 20 mM Tris-Hcl pH 7.5, 20 mM DTT, 1 mM TGIRT-III enzyme, 500 μg/ml of Oligo-dT or Random primer and RNAse inhibitor cocktail). The solution was pre-incubated at room temperature for 30 min, then added dNTPs (final concentration 1 mM). The reverse transcription was done at 60°C for 45 min. In order to inactivate the enzyme, the reaction was incubated with NaOH (250 mM final concentration) for 3 min at 95°C and then neutralized with HCl (250 mM final concentration) at room temperature. The cDNA was then cleaned up with a GeneJET PCR purification kit (Thermo Scientific). For newly synthesized RNA, cells were treated with ethynil uridine and neo-synthesized RNAs were purified with Click-iT Nascent RNA Capture Kit (Invitrogen) according to the manufacturer’s instructions.

### Real-time quantitative PCR

RT-qPCR analyses were performed using Taqman probes (SsoAdvanced Universal Probes Supermix, Biorad) or SYBR green (SsoAdvanced™ Universal SYBR® Green Supermix, Biorad). Analyses were conducted via the CFX Connect Real-Time detection System (Biorad) and the expression level was determined by using CFX Manager Software (Biorad). The relative expression of different transcripts was normalized to β-actin mRNA or β-glucuronidase (GUS) mRNA as endogenous controls. RT-qPCR primer and probes sequences are listed in Additional file 3: Table S2.

### Digital PCR

Digital PCR was performed using the QuantStudio 3D PCR system (Applied Biosystem) according to the manufacturer’s instructions. Reactions were prepared using specific primers and probes for the target detection as reported in Additional file 3: Table S2. The equivalent amount of 100 ng of retrotranscribed RNA was used for the absolute quantification of 3′ EIF4A1 snoRTs fragment and full-length EIF4A1 snoRT; results were normalized against the GUS housekeeping gene.

### 5′RACE

Mapping of the 5′ end of the snoRT transcripts was performed by rapid amplification of cDNA ends (RACE) using the 5′/3′ RACE kit 2nd generation (Roche Diagnostics GmbH) following the manufacturer’s instructions. In order to obtain a less complex sample to analyze, we performed the primer extension reaction using RNA purified from the cytoplasmic RIP with an anti-dyskerin antibody. Primer extension for first-strand cDNA synthesis was performed using reverse primers positioned on the 3′ end of the retained intron. After poly-(A) tailing of the cDNA, in order to amplify all the dA-tailed cDNA, a PCR amplification was performed using a specific oligo dT-Anchor primer, provided by the kit, and a second nested reverse primers, with the AccuPrime™ Taq DNA Polymerase System (Thermo Scientific). A second PCR was performed using a specific PCR Anchor primer, provided by the kit, and third nested reverse primers. The purification of PCR amplicons was performed using the QIAquick PCR purification kit (Quiagen Inc.) following the manufacturer’s instructions and the purified product was sequenced using the last nested reverse primers. The 5’ end was characterized by aligning the sequence on the specific gene. Primer sequences are listed in Additional file 3: Table S2.

### Western blotting

Protein lysates were diluted in Laemmli loading dye (2% SDS, 8% glycerol, 62.5 mM Tris-HCl pH 6.8, 0.005% bromophenol blue, and 2% β-mercaptoethanol) and separated on a 10% polyacrylamide gel by SDS-PAGE (TGX Stain-Free™ FastCast™ Acrylamide Solutions; Biorad) or 4-15% polyacrylamide gel (Mini-PROTEAN® TGX Stain-Free™; Biorad). Proteins were transferred to nitrocellulose membranes (Amersham Protran 0.2um nc), blocked, and probed overnight at 4°C with appropriate antibodies. Detection was performed with appropriate HRP-conjugated secondary antibodies and chemiluminescent detection was performed on a ChemiDoc XRS+ Imaging System (Biorad) using ImageLab v5.1.1 (Biorad). The Stain-Free system allows protein loading and blotting control, especially for RIP and polysome protein gels. The following antibodies were used: anti-dyskerin (Santa Cruz Biotechnology, sc-373956), anti-Vimentin (Cell Signaling, 5741S), anti-GAPDH (Sigma-Aldrich, G8795), anti-b-Actin (Sigma-Aldrich, A2228), Lamin B1 (Santa Cruz Biotechnology, sc-6216), anti-c-Myc (Cell Signaling, 5605S), anti-pEGFR (Cell Signaling, 53A5), anti-c-Jun (Cell Signaling, 9165S), anti-PARP (Cell Signaling, 9542S), anti-NHP2 (Santa Cruz Biotechnology, sc-398430), anti-NOP10 (Cusabio, CSB-PA873610LD01HU), anti-GAR1 (Proteintech, 11711-1-AP) anti-RPS14 (Santa Cruz Biotechnology, sc-68873), anti-RPL5 (Bethyl, A303-933A), anti-EIF4GI (Cell Signaling, 2858S) anti- puromycin (Merk, MABE343).

### Polysome fractionation

For standard polysome fractioning, cultured MCF7 cells were treated with 100 μg/ml cycloheximide (CHX) while for testing whether transcripts are truly associated with polysomes, cultured MCF7 cells were treated with 100 μg/ml puromycin. Cells were incubated for 20 min at 37°C and washed on ice twice with cold PBS containing 100 μg/ml CHX or 100 μg/ml puromycin. Cells were scraped and lysed in Cytoplasm Lysis Buffer for cytoplasmic sub-fractioning (as described above) with the addition of CHX or puromycin. The lysate was centrifuged at 14,000 g for 10 min at 4°C. Lysates containing from 700 μg to 1000 μg of total proteins were layered onto a chilled sucrose gradient (15–50% or 10–30%) in low-salt buffer (LSB) (20 mM Tris-HCl pH 7.5, 10 mM NaCl, 3 mM MgCl_2_, 0.4% IGEPAL, 50 mM sucrose, and RNAse inhibitors) containing 100 μg/ml CHX. Gradients were centrifuged at 36,000 RPM for 2 h at 4°C in a SW41 rotor (Beckman Coulter) and then fractions were collected using a gradient collector (Teledyne ISCO gradient station) coupled with UV detector to continue monitoring the absorbance at 254 nm. For mRNA sequencing, 10% of each fraction was pooled to reconstitute the total mRNA, while the remaining polysomal fractions were pooled together. For 10–30% polysome profiling, one half of each fraction was used for protein purification and the other half for RNA purification. Proteins were recovered using acetone precipitation: the same volume of 100% ice-cold acetone and 1/10^th^ of TCA were added to each fraction. The samples were placed at −80°C overnight and then centrifuged at 16,000 g for 10 min at 4°C. The resulting pellets were washed three times with 1 ml of 100% ice-cold acetone in order to remove TCA residuals and dried at RT for 5 min. Pellets were finally resuspended in a Laemmli loading dye. RNA was purified as described above.

### Ribosome purification

Human ribosomes from MCF7 and MDA-MB 231 cells were purified by lysing cell pellets via the addition of 2 packed cell volumes of 10 mM Tris-HCl, pH 7.5, 10 mM NaCl, 3 mM MgCl2, and 0.5% IGEPAL for 10 min at 4°C. The lysate was centrifuged at 20,000 g for 10 min at 4°C to isolate the cytoplasmic fraction from nuclei and mitochondria. Highly purified ribosomes (high salts) were obtained as previously described [53]. Briefly, 500 μl of lysate was incubated 10 min at 37°C to enable ribosomes to complete translation and detach from the mRNAs they were translating. Then the cytoplasmic lysate was layered on a discontinuous sucrose gradient in stringent conditions (top-half gradient: 1.0 M sucrose 30 mM Hepes/KOH, pH 7.5, 2 mM magnesium acetate, 1 mM DTT and 70 mM KCl; bottom-half: 0.7 M sucrose, 30 mM Hepes/KOH, pH 7.5, 2 mM magnesium acetate, 1 mM DTT and 0.5 M KCl) and ribosome were precipitated by centrifuging samples for 16 h at 160,000 g at 4°C. For less stringent purification (low salt), 500 μl of lysate was layered on a single sucrose cushion (1 M sucrose, 10 mM Hepes pH 7.5, 10 mM potassium acetate, 1 mM magnesium acetate, and 1mM DTT) and then centrifugated at the same condition of high salt purification. Ribosomes were washed twice with 10 mM Tris/HCl pH 7.5, 2 mM magnesium acetate, and 100 mM ammonium acetate, and resuspended in the same buffer. Ribosome concentration was calculated from the absorbance at 260nm (1 mg/ml ribosome = 12.5 A260).

### Overall protein synthesis rate

To assess the overall protein synthesis rate, cells were treated with 1 μg/ml of puromycin for 15 min and after another 45 min the cells were lysed as described above. Because only newly synthesized proteins incorporate puromycin, the synthesis rate can be evaluated by Western blotting using an anti-puromycin antibody.

### Cell proliferation assay

Cell proliferation assay were performed on EIF4A1-snoRT siRNA silenced MCF7 cells as described above. At specific time-points cells were fixed in formalin and then stained with crystal violet (0.1 % crystal violet in 20% methanol). After staining, cells were washed three times with PBS and destained with 10% acetic acid, and the absorbance of the crystal violet solution was measured at 590 nm.

### Patients’ material

One hundred twenty breast carcinomas were selected for EIF4A1 mRNA expression determination from a series of consecutive patients who underwent surgical resection for primary breast carcinoma at the Surgical Department of our institution, on the sole basis of frozen tissue availability. Some of the cases were obtained from a previous study [2]. Data on patients’ survival, tumor histological classification, estrogen and progesterone receptor status were obtained as described elsewhere [2]. Data collection was subject to the availability of specific patient information or surgical tissues.

### Experiments with mice

All animal work was approved by Bologna University’s Institutional Animal Care and Use Committee in accordance with national guidelines and standards (protocol approval reference No. 204/2016-PR). Six-week-old female BALB/c nude mice were purchased from Charles River (Charles River Laboratories Italia s.r.l.). The mice were maintained in a specific pathogen-free facility, on a 12-h light-dark cycle at 21°C. To perform the breast cancer xenografts, 5 million stably dyskerin interfered MCF7 cells and the relevant control cell line were injected subcutaneously into both flanks of anesthetized mice (10 mice per cell line). No estradiol supplement was administered to the mice [54]. The mice were weighed once a week, and the tumor growth was monitored weekly. The animals were euthanized at post-injection week 33, by an anesthetic overdose according to the approved experimental protocol.

### mRNA sequencing

RNA purified from polysomal fractioning (the reconstituted total mRNA and pooled polysomal RNA) was tested for quality using the RNA 6000 nano kit (Agilent) on a Bioanalyzer 2100 (Agilent).

Poly-A enriched, strand-specific RNA libraries were generated with the TruSeq mRNA Stranded sample preparation kit (Illumina) starting from 1 μg of RNA from each fraction (total and polysomal).

Briefly, RNA was subjected to poly(A) selection using Magnetic Oligo-dT Beads. Poly(A^+^) RNA was partially degraded by incubating in Fragmentation Buffer at 94°C for 4 min. After first- and second-strand cDNA synthesis using random primer, end repair and A-tailing modification, Illumina Truseq sequencing adaptors were ligated to cDNA ends. cDNAs were amplified by 15 cycles of PCR reactions and subsequently purified by AMpure XP beads (Beckman). Each individual library was quantified, and quality controlled using a Qubit Fluorometer (Thermo Scientific), LabChip GX (Perkin Elmer). After libraries equimolar pooling, the final pool was quantified by RT-qPCR (KAPA and Biorad). The adaptor-tagged pool of libraries was loaded on an Illumina Hiseq2500 high throughput flowcell (PE50 chemistry) for cluster generation and deep sequencing. Reads were filtered by quality and trimmed, and adapters removed (minimum quality 30, minimum length 36 nt) with Trimmomatic [55]. Reads were then aligned to the hg38 genome with STAR [56], using the Gencode v28 (http://www.gencodegenes.org/releases/) annotation to quantify genes. Transcripts were aligned and quantified with Salmon [57] on the same annotation. DESeq2 [58] was then used to call Differentially Expressed Genes (DEGs) between conditions (shDKC1 vs control) at the total and polysomal level, using a 0.05 threshold on the adjusted *p*-value (Data are available on Additional file 4: Table S3 Supplementary Table S3).

Exon analysis was performed on aligned reads with DEXseq [59], using an adjusted *p*-value threshold of 0.05 to compare conditions at the total and polysomal level.

The functional enrichment analysis on Gene Ontology annotations was performed by the topGO R package [60], using a BH-adjusted *p*-value threshold of 0.05 (data are available on Additional file 5: Table S4).

### RIP-seq

RIP assay was performed as described above, and the cytoplasmic RNA purified from RIP was tested for quality using the RNA 6000 nano kit (Agilent) on a Bioanalyzer 2100 (Agilent), while 10 ng of the extracted RNA was used for fragmentation at 94°C for 4 min. RNA libraries were generated using the SMART-Seq Stranded Kit (Takara). This kit incorporates SMART® cDNA synthesis technology [61] and generates Illumina-compatible libraries via PCR amplification, thus avoiding the need for adapter ligation and preserving the strand orientation of the original RNA. The Ribosomal cDNA was depleted by a ZapR-mediated process, in which the library fragments originating from rRNA and mitochondrial rRNA are cleaved by ZapR in the presence of mammalian-specific R-Probes. Library fragments originating from non-rRNA molecules were enriched via a second round of PCR amplification using Illumina-specific primers and, subsequentially, purified. Each individual library was quantified and quality-controlled using a Qubit Fluorometer (Thermo Scientific), LabChip GX (Perkin Elmer). Equal numbers of cDNA molecules from each library were pooled and the final pool was purified once more in order to remove any free primer. Following a final RT-qPCR quantification (KAPA and Biorad), the pool was loaded on a Hiseq2500 rapid run flow-cell and run in a PE50 chemistry. Reads were pre-processed (quality threshold Q30, minimum length 36nts, adapters removed) using TrimGalore (https://www.bioinformatics.babraham.ac.uk/projects/trim_galore/), then aligned to the hg38 genome with STAR [56], using the Gencode v28 (http://www.gencodegenes.org/releases/) annotation to quantify genes. Transcripts were aligned and quantified by Salmon [57] on the same annotation. Gene read counts were normalized by library size. RIP fold-enrichment and *p*-value were computed for each condition using DESeq2 [58] as (RIP/INPUT) or (IGG/INPUT). Genes and transcripts which became significantly enriched (adjusted *p*-value <= 0.05) in the RIP/INPUT and not in the corresponding IGG/INPUT were considered to be dyskerin targets. The functional enrichment analysis of dyskerin targets was performed as previously described (Data are available on Additional file 6: Table S5).

### Analysis of the sequence motifs

The sequence of the 701 transcripts found to be bound by DKC1 in our cytoplasmic RNA immunoprecipitation was retrieved with the biomaRt R package [62] and provided it as input to STREME [63], set to find motifs with *p*-value < 0.05 and length between 8 and 15 nucleotides. ENCODE DKC1 eCLIP data was retrieved from the ENCODE Data Portal (encodeproject.org) and sequences of binding sites extracted through the biomaRt R package [62]. Matches to the motifs identified by STREME were obtained with FIMO v5.4.1, using a threshold of 1E-04 on the matches *p*-value and allowing matches only on the sense strand. STREME [63] was then run on these sequences with the same parameters listed above. ANANNA and ACA sites occurrences were predicted with FIMO [63]. Finally, Pearson correlation between pairs of RIP and eCLIP motifs was computed with the TFBSTools R package [64] and reported only for pairs with correlation above 0.5.

### Analysis of NMD sensitive transcripts on independent dataset

To identify whether some of the transcripts found enriched by RIP-seq analysis are sensitive to NMD, we analyzed a public dataset of two-replicate comparison between siRNA silencing of UPF1 and ctrl on HeLa cells (SRA Accession ID SRP063462, available at: 10.6078/D1H019). The analysis was performed on aligned reads with DEXseq, using an adjusted *p*-value threshold of 0.05 (as described above) and a padj threshold of log2FC = 1.

### Survival analysis on independent dataset

For the analysis of survival curves on public independent dataset we used the GEPIA2 [39] analysis tool (http://gepia2.cancer-pku.cn/) to obtain the overall survival (OS) of 1085 TCGA breast invasive carcinomas patients. The analysis was performed with a median group cutoff to split the high-expression and low-expression cohorts of the identified signature. The same tool was used for the analysis of the correlation expression of the identified signature against DKC1.

### Statistical analysis

Statistical analyses (tests, number of replicates, and two-sided *p*-values) are indicated in the corresponding figures or figure legends.

## Supplementary Information

Additional file 1: Supplementary Figures S1-S5. 

Additional file 2: Table S1. RIP-seq transcripts correspondences.

Additional file 3: Table S2. Primers and probes.

Additional file 4: Table S3. RNA-seq. Transcript-Based Analysis.

Additional file 5: Table S4. RNA-seq. Exon-Based Analysis.

Additional file 6: Table S5. RIP-seq Analysis.

Additional file 7. Uncropped Western Blot images.

Additional file 8. Review history.

## Data Availability

The RNA-seq and RIP-seq datasets were deposited in the Gene Expression Omnibus (GEO) under the accession number GSE161481 [65].
