# Supplementary material for: Human dyskerin binds to cytoplasmic H/ACA-box-containing transcripts affecting nuclear hormone receptor dependence
Source: Genome Biol. 2022 Aug 22;23:177. doi: 10.1186/s13059-022-02746-3 (PMC9394076; doi:10.1186/s13059-022-02746-3)
Supplement: Supplementary file 1 — Additional file 1: Supplementary Figures S1-S5. [file 13059_2022_2746_MOESM1_ESM.docx]

**Human dyskerin binds to cytoplasmic H/ACA-box-containing transcripts affecting nuclear hormone receptor dependence**

Federico Zacchini^1,2^, Giulia Venturi^1,2^, Veronica De Sanctis^3^, Roberto Bertorelli^3^, Claudio Ceccarelli^4,1^, Donatella Santini^4^, Mario Taffurelli^5,6^, Marianna Penzo^1,2^, Davide Treré^7,1^, Alberto Inga^3^, Erik Dassi^3^, Lorenzo Montanaro^1,7^*

^1^ Dipartimento di Medicina Specialistica, Diagnostica e Sperimentale (DIMES), Alma Mater Studiorum - Università di Bologna, Bologna, I-40138 - Italia

^2^ Centro di Ricerca Biomedica Applicata – CRBA, Università̀ di Bologna, Policlinico di Sant’Orsola, Bologna, I-40138 - Italia

^3^ Dipartimento di Biologia Cellulare, Computazionale e Integrata (CIBIO), Università di Trento, I-38123 Trento - Italia

^4^ Unità Operativa di Anatomia Patologica, IRCCS Azienda Ospedaliero-Universitaria di Bologna, Via Albertoni 15, I-40138 Bologna - Italia

^5^ Unità Operativa di Chirurgia Generale, IRCCS Azienda Ospedaliero-Universitaria di Bologna, Via Albertoni 15, Bologna I-40138 - Italia

^6^ Dipartimento di Scienze Mediche e Chirurgiche (DIMEC), Alma Mater Studiorum - Università di Bologna, Bologna, I-40138 - Italia

^7^ Departmental Program in Laboratory Medicine, IRCCS Azienda Ospedaliero-Universitaria di Bologna, Via Albertoni 15, Bologna, I-40138 - Italia

* To whom correspondence should be addressed. Tel: +39 0512083042; Fax: +39 051306861; Email: Lorenzo.montanaro@unibo.it


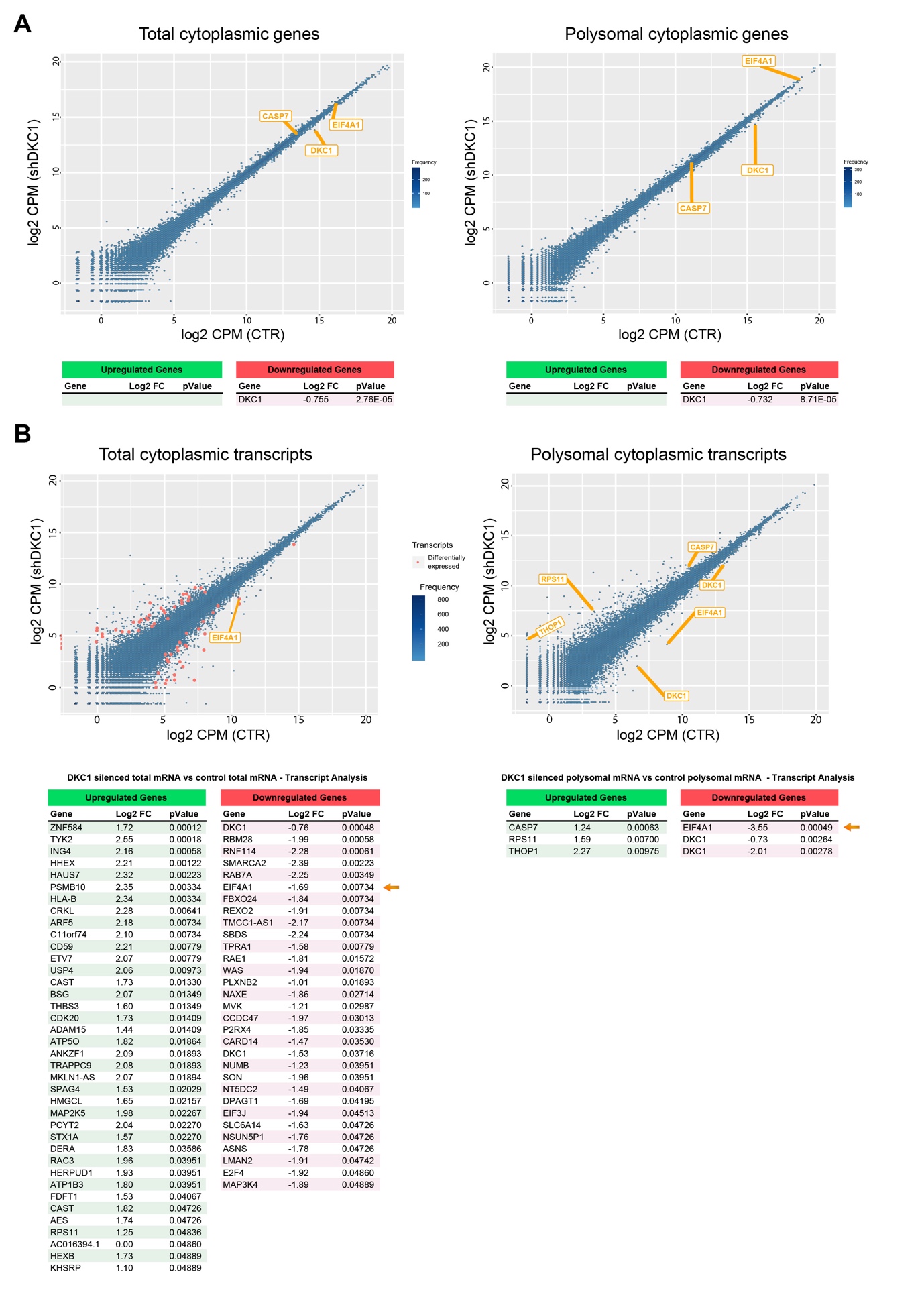


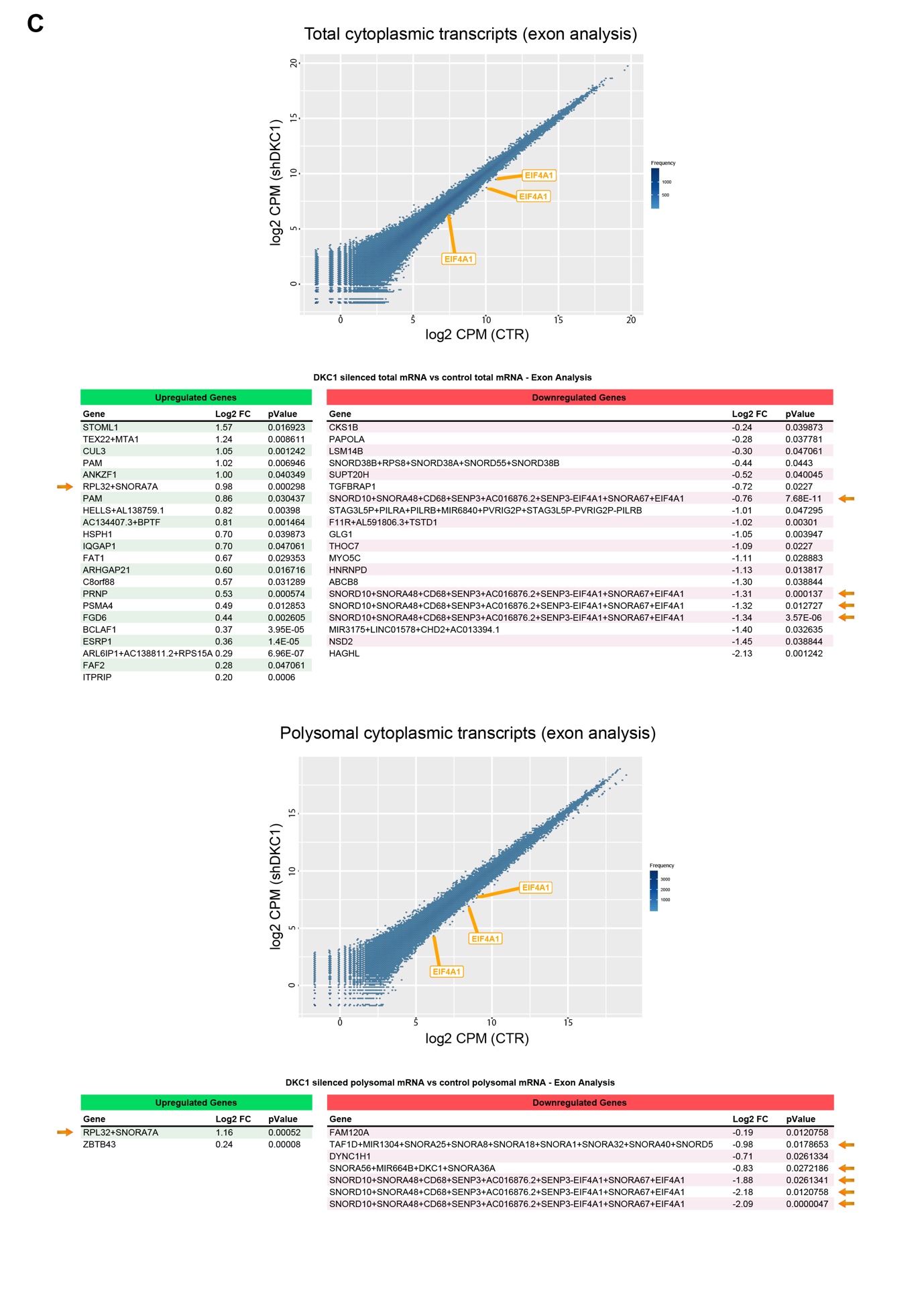


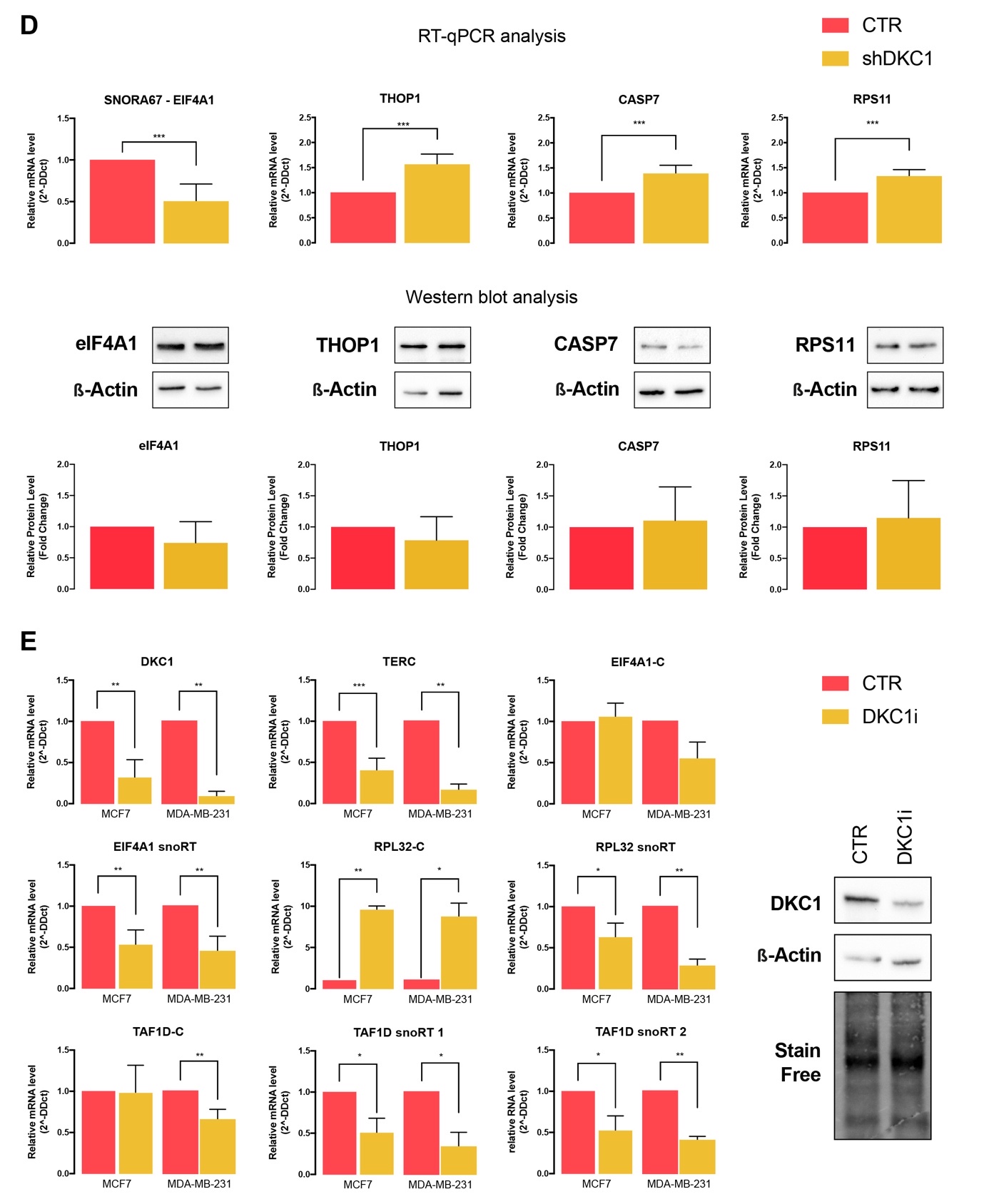


**Fig S1**, related to Figure 1. **Differential expression analysis of total cytoplasmic and polysomal recruited species and validation by RT-qPCR.**

**A-B-C** Count-based differential expression analysis of total cytoplasmic (A), transcripts (B) (the same images as Figure 1C), and transcripts obtained from exon analysis (C). Data are reported for both total cytoplasmic and polysomal RNA analysis. Differentially expressed transcripts are depicted as red dots, while transcript gene names of interest are highlighted. Given the small number of differentially expressed transcripts in the polysomal transcript analysis, those are all individually labelled instead of being shown as red dots. Below every plot there is a table with all the corresponding regulated gene names. Orange arrow highlight H/ACA snoRTs. For the gene or transcript ID see Supplementary Tables S2 and S3 file.

**D** Validation by RT-qPCR quantification of the specific transcripts differentially recruited on polysomes on the same MCF7 cytoplasmic cell lysate used for the RNA-seq (top). Representative Western blotting analysis and densitometric analysis of the corresponding proteins of MCF7 cytoplasmic cell lysate (bottom). The means from three biological replicates (n = 3) are shown; error bars represent SD. Paired Student’s t tests were performed relative to the controls.

**E** RT-qPCR quantification of the transcripts found to be differentially recruited on the polysomes observed in MCF7 and MDA-MB 231 total cell lysates after dyskerin KD using siRNA. A representative Western blot analysis image (right) of dyskerin levels after DKC1 mRNA RNAi in MCF7 cells is also shown. DKC1 and TERC are used for dyskerin silencing control. The means from three biological replicates (n = 3) are shown; error bars represent SD. Paired Student’s t tests were performed on the controls.

*p < 0.05, **p < 0.01, ***p < 0.005, ****p < 0.001.


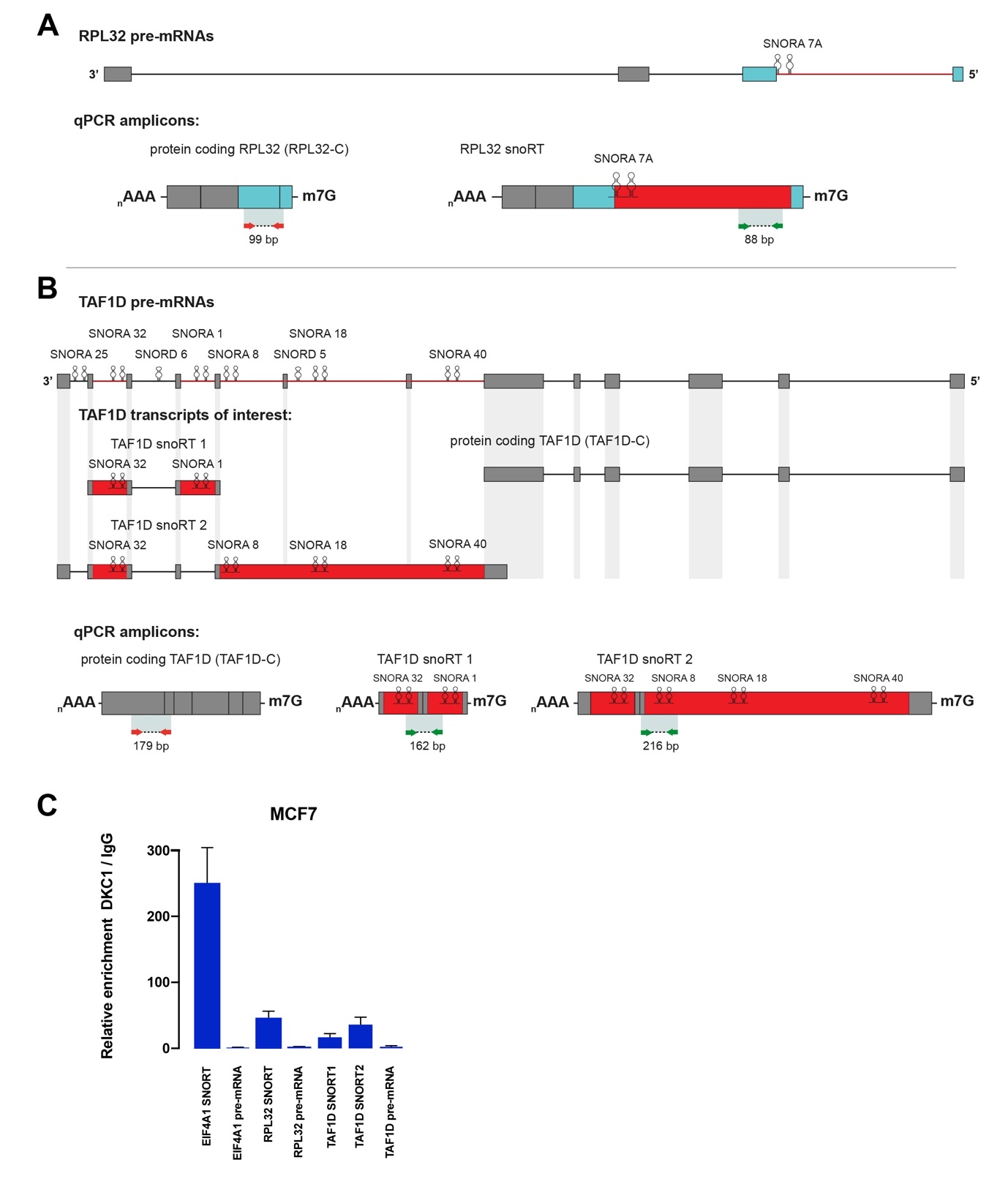


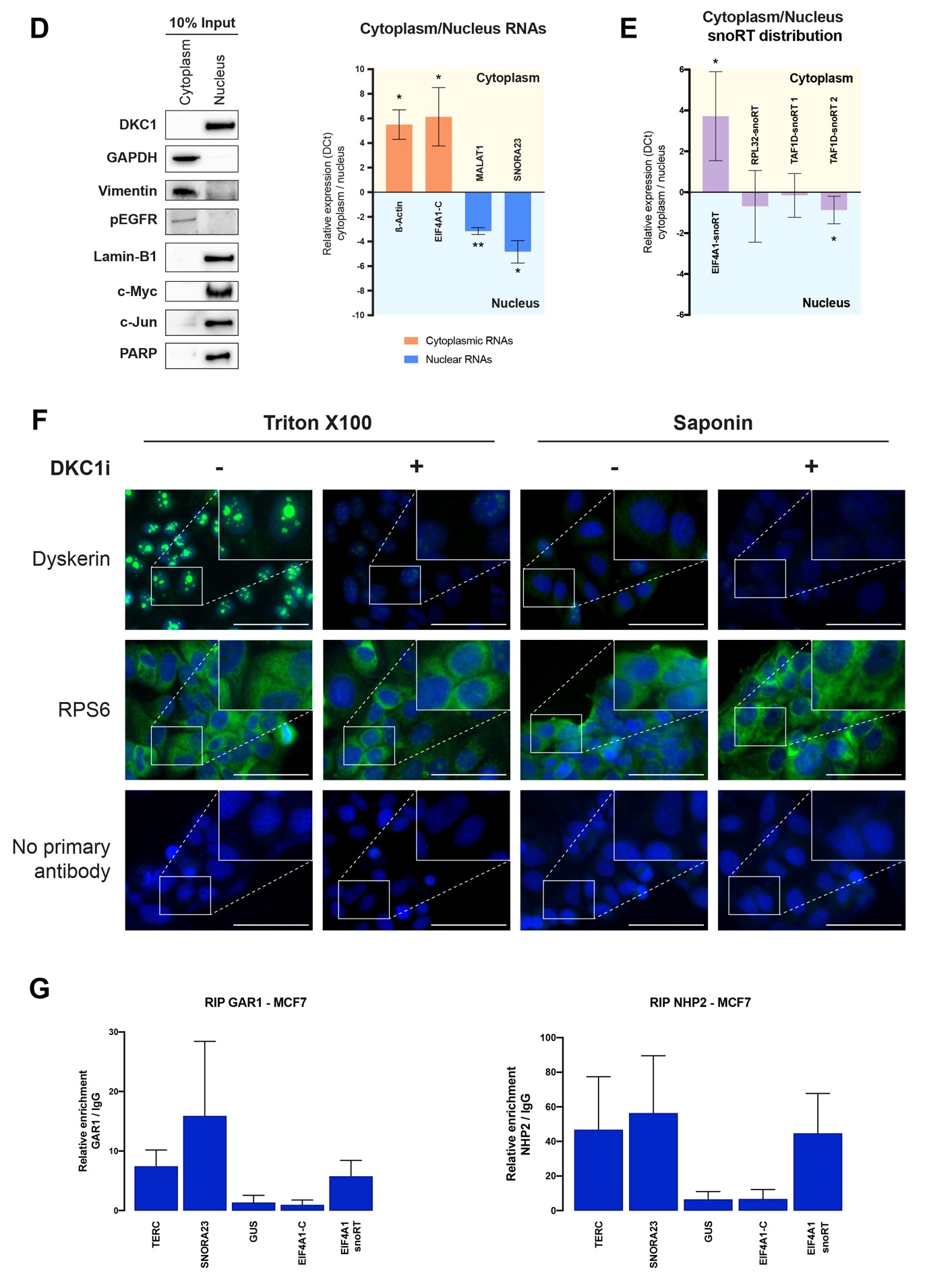


**Fig S2,** related to Figure 2**. Analysis of cytoplasmic snoRTs**

**A** To-scale schematic overview of the RPL32 pre-mRNA. Introns are depicted as lines connected to exons. SNORA7A is shown, and its intron is depicted as a red line in pre-mRNA or as a red box in RPL32 snoRT. Exons flanking intron-containing*-*SNORA7A sequence are depicted as blue boxes. The RT-qPCR amplicons are shown below the mRNA boxes, between arrows. The amplicon between red arrows identifies the protein coding mRNA, while the amplicon between green arrows identifies every RPL32 snoRT. Primer sequences are listed in Supplementary Tables S1. m7G: cap; AAAn: poly(A) tail; P: monophosphate.

**B** To-scale schematic overview of the TAF1D pre-mRNA. Introns are depicted as lines connected to exons. SNORA25, SNORA32, SNORA1, SNORA8, SNORA18 SNORA40, SNORD6, and SNORD5 are shown. Intron-retaining SNORA sequences of interest are depicted either as a red line in pre-mRNA or as a red box in TAF1D transcripts of interest. RT-qPCR amplicons are indicated. The amplicon between red arrows identifies the protein coding mRNA, while the amplicon between green arrows (different primers) specifically identifies the two transcripts shown in the figure. Primer sequences are listed in Supplementary Tables S1. m7G: cap; AAAn: poly(A) tail; P: monophosphate.

**C** RT-qPCR analysis of snoRT pre-mRNAs performed on samples obtained by RNA immunoprecipitation of dyskerin from MCF7 total cell lysate. The assessment of snoRTs level is the same depicted in figure 2B while the level of each pre-mRNA was obtained with specific primers which target other introns not containing any snoRNA sequence. The means from three biological replicates (n = 3) are shown; error bars represent SD. Paired Student’s t tests were performed relative to controls.

**D** Isolation of cytoplasmic and nuclear fractions. Western blotting analysis (left) of cytoplasmic and nuclear fractions obtained from MCF7 cells. Vimentin and pEGFR was used for anchored proteins in the cytoplasm while lamin-B1 was used for anchored proteins in the nucleus. GAPDH was used for soluble proteins in the cytoplasm, while c-Myc, c-Jun, and PARP1 were used for soluble proteins in the nucleus. The right panel show the relative expression (Dct - cytoplasm vs nucleus) of RNA species known to localize prevalently either in the cytoplasm or in the nucleus. The means from three biological replicates (n = 3) are shown; error bars represent ± SD. Unpaired Student’s t test were performed compared to zero (no difference in distribution).

**E** Cytoplasmic and nuclear distribution of snoRTs. RT-qPCR of snoRT subcellular distribution (Dct - cytoplasm vs nucleus). The means from four biological replicates (n = 4) are shown; error bars represent ± SD. Unpaired Student’s t test were performed compared to zero (no difference in distribution).

**F** Immunofluorescence images of MCF7 cells treated with control or DKC1 siRNAs. Cells were permeabilized with Triton X-100 or saponin in order to obtain the permeabilization of the cell membrane and nuclei or for the cell membrane only respectively. Cell were stained with an antibody anti-dyskerin, anti-RPS6 (used as cytoplasmic marker) or with no primary antibody (used as negative control). Nuclei were visualized with DAPI staining. The scale bar indicates 64 µm.

**G** RNA immunoprecipitation analysis of GAR1 and NHP2 from MCF7 total cellular lysates. RT-qPCR analysis of RIP with anti-GAR1 antibody and anti-NHP2 antibody from MCF7 total cellular lysates. The analysis was performed on the known dyskerin targets (TERC, SNORA23), a known off-target (GUS), and EIF4A1 canonical or snoRT isoform. Results are expressed as the fold change of GAR1 or NHP2 immunoprecipitation RNA level against immunoprecipitation with IgG. Data are shown as means ± SEM. n=3 biological replicates were performed for each experiment. Unpaired Student’s t tests were performed on respective controls IgG.


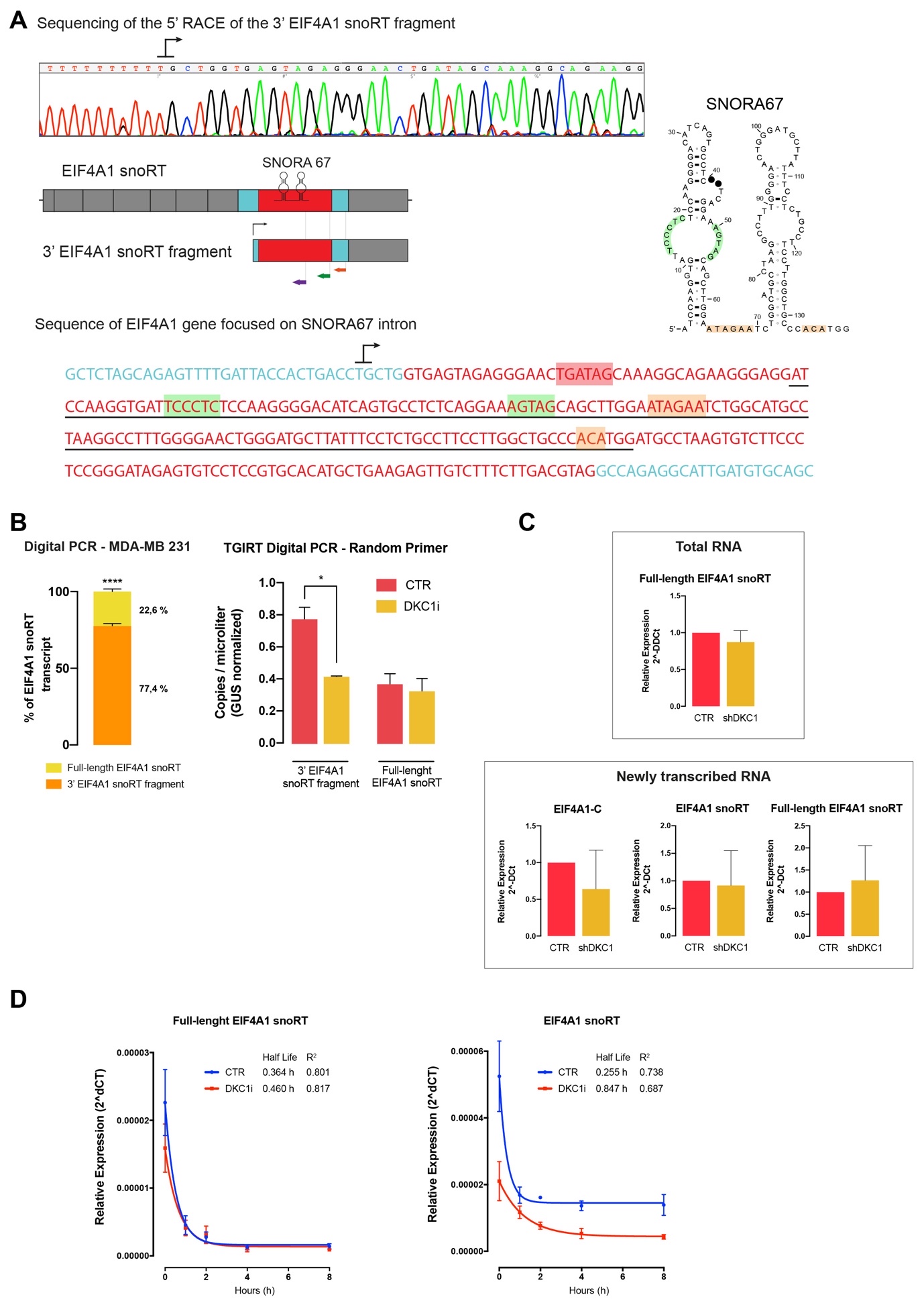


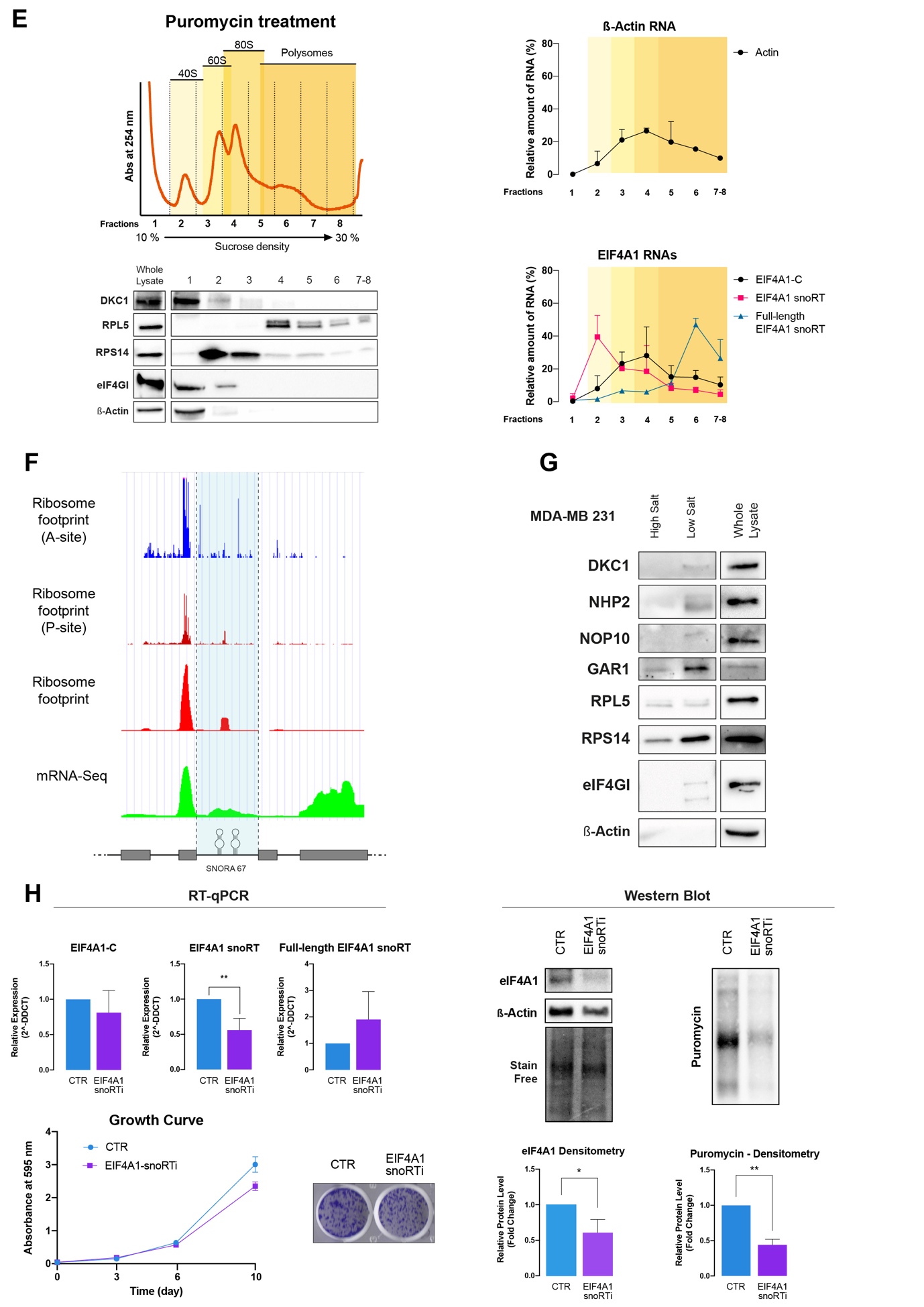


**Fig S3**, related to Figure 3. **The dyskerin-bound EIF4A1 snoRT is truncated at its 5’ end and interacts with ribosomes in the cytoplasm.**

**A** Sequencing of the EIF4A1 snoRT fragment by 5’ RACE. The top panel shows the sequence of the 5’ portion of the transcript. The arrow indicates the two possible starting bases after the poly-(T) tail added during the 5’ RACE. The middle panel shows the scheme of the EIF4A1 snoRT, the 3’ EIF4A1 snoRT fragment and the scheme of the SNORA67. The lower panel shows the sequence of the EIF4A1 gene, focused on SNORA67 intron. Exons flanking intron-containing-SNORA67 sequence are depicted as blue boxes or sequences, while the retained intron is depicted as a red box or sequence. The colored arrows represent the primers used for the 5’RACE (orange primer: “EIF4A1 snoRT dPCR RV”; green primer: “EIF4A1 snoRT RV”; purple primer: “EIF4A1 snoRT nest”). The sequence of SNORA67 is underlined. The snoRNA sequences complementary to the target RNA are highlighted in green while the H box and ACA box sequences are highlighted in orange. The “in-frame” STOP codons are highlighted in red. The black arrow indicates the two possible starting bases of the EIF4A1 snoRT fragment.

**B** Left: Percentage representation of EIF4A1 snoRT obtained by digital PCR absolute quantification in MDA-MB 231 cells. Data are shown as the percentage of EIF4A1 snoRT after normalization with GUS housekeeping transcript. Right: digital PCR absolute quantification of cDNA obtained by TGIRT reverse transcriptase using Random Primers (RP) in MCF7 cells after siRNA silencing of DKC1. Data are shown as copies/microliter after normalization on GUS housekeeping transcript. The means from three biological replicates (n = 3) are displayed, error bars represent SD. Paired Student’s t tests were performed relative to controls.

**C** Top: RT-qPCR analysis of the full-length EIF4A1 snoRT of total RNA from MCF7 lysates after shDKC1 silencing. Bottom: RT-qPCR analysis of the EIF4A1 transcripts of interest from newly transcribed RNA from MCF7 lysates after shDKC1 silencing. The means from three biological replicates (n = 3) are displayed, error bars represent SD. Paired Student’s t tests were performed relative to controls.

**D** mRNA stability assay of EIF4A1 snoRT species after actinomycin D treatment. MCF7 cells with stable depletion of dyskerin and the relative controls were seeded at 70% confluence and incubated with actinomycin D (10 μg/ml) added to the medium. Cells were harvested at 0, 1, 2, 4, 8 h after treatment and RNA was extracted. Data are shown as relative expression (2^dCT) to the relatively stable rRNA 18S. The means from three biological replicates (n = 3) are shown, error bars represent SEM.

**E** Polysome profiling analysis after puromycin treatment. Top left: representative polysome profile obtained by 10-30% sucrose density gradient centrifugation from MCF7 cells. Puromycin treatment prevents ribosome translocation during the elongation stage, resulting in a low number of polysomes obtained. The portions of the profile referring to the different ribosomal subunits are highlighted. Bottom left: distribution of dyskerin and control proteins across the gradient fractions analyzed by Western blotting with specific antibodies. Right: Distribution of transcripts of interest after RNA purification from gradient fractions obtained by RT-qPCR. Results are expressed as the fraction (%) of the total amount of the transcripts contained in the lysate. Data are shown as means ± SEM of two different biological replicates.

**F** Ribosome profile of global aggregate data obtained on UCSC Genome Browser database using GWIPS-vis tool. Only the portion near the SNORA 67 is shown. SNORA 67 intron is highlighted in blue.

**G** Ribosome purification: Western blotting analysis of purified ribosomes from MDA-MB 231 cells shows a co-purification of all pseudouridine-RNP complex (DKC1, NHP2, NOP10, GAR1). RPL5 and RPS14 are shown as the positive control for the ribosomal purification, while eIF4G is used as the control for ribosome interacting factors.

**H** EIF4A1 snoRT siRNA silencing (EIF4A1 snoRTi) of MCF7 cells. Top left: RT-qPCR analysis of the EIF4A1 transcripts of interest. The means from three biological replicates (n = 3) are displayed, error bars represent SD. Paired Student’s t tests were performed relative to controls. Bottom left: crystal violet growth curve analysis. The means from three biological replicates (n = 3) are displayed, error bars represent SD. A representative crystal violet staining is shown. Right: representative images of Western blotting analysis and respective densitometric analysis of eIF4A1 protein (left) and puromycin (right). Puromycin detection was performed for assessing overall protein synthesis rate. The means from three biological replicates (n = 3) are displayed, error bars represent SD.

*p < 0.05, **p < 0.01, ***p < 0.005, ****p < 0.001.


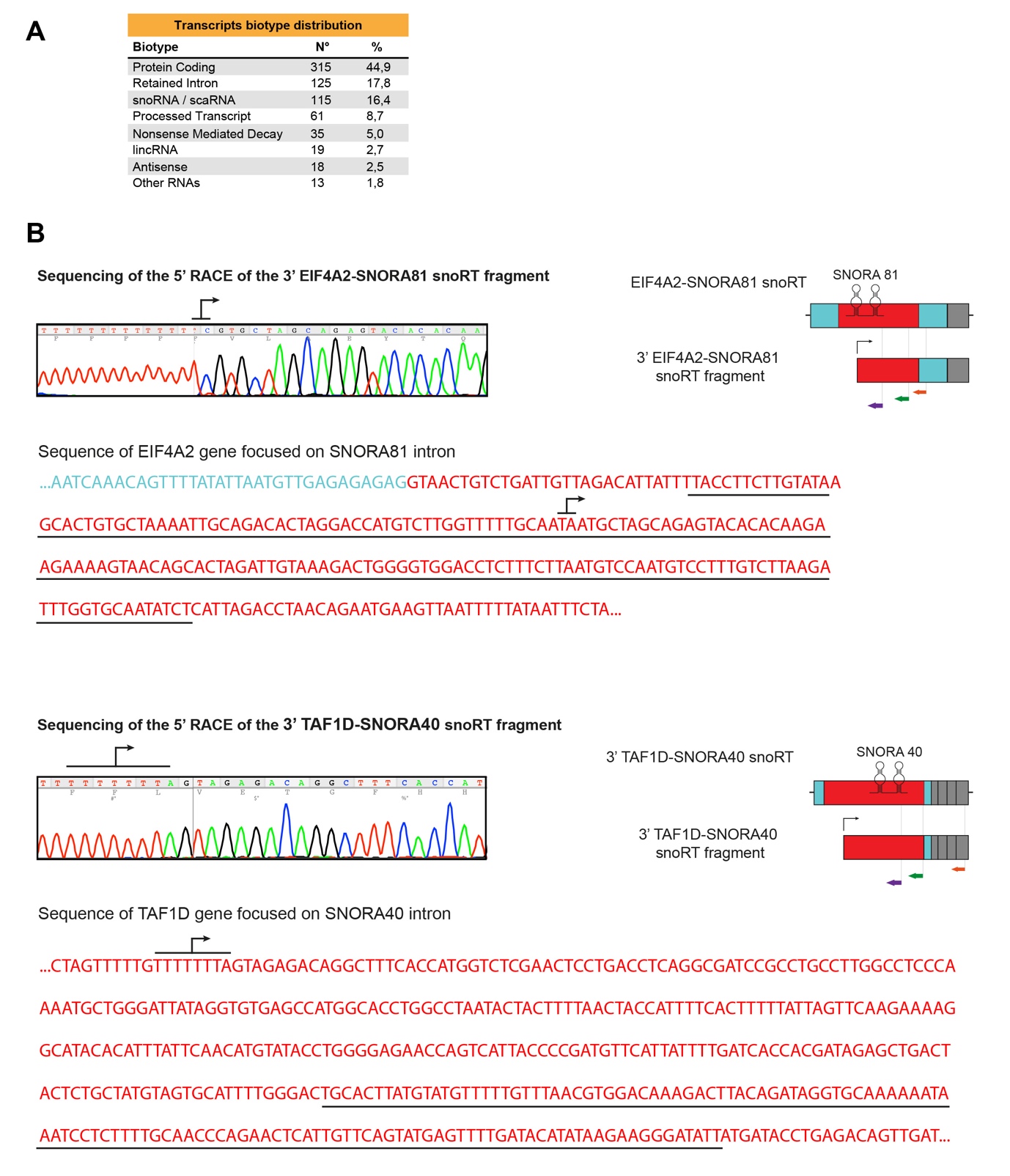


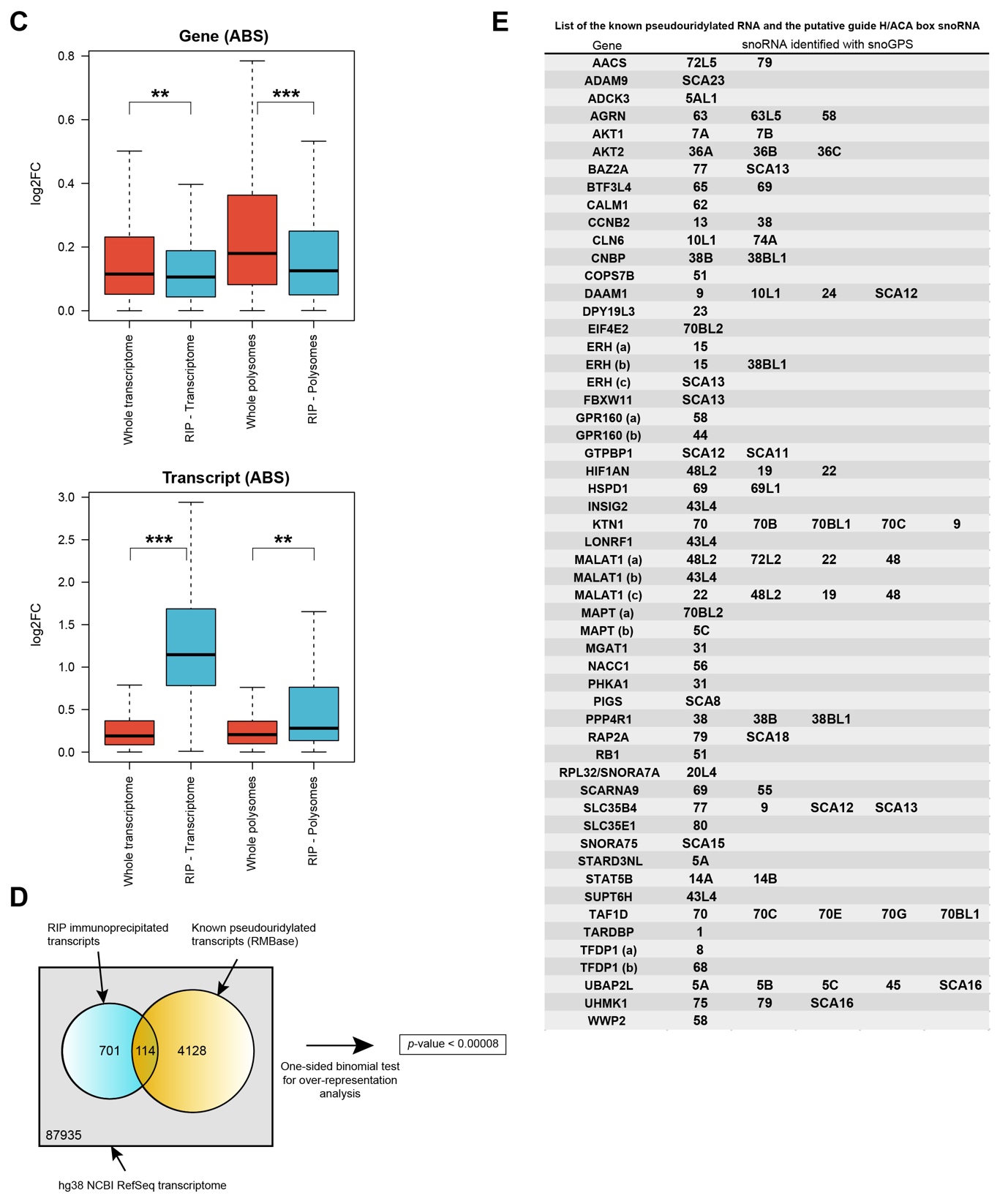


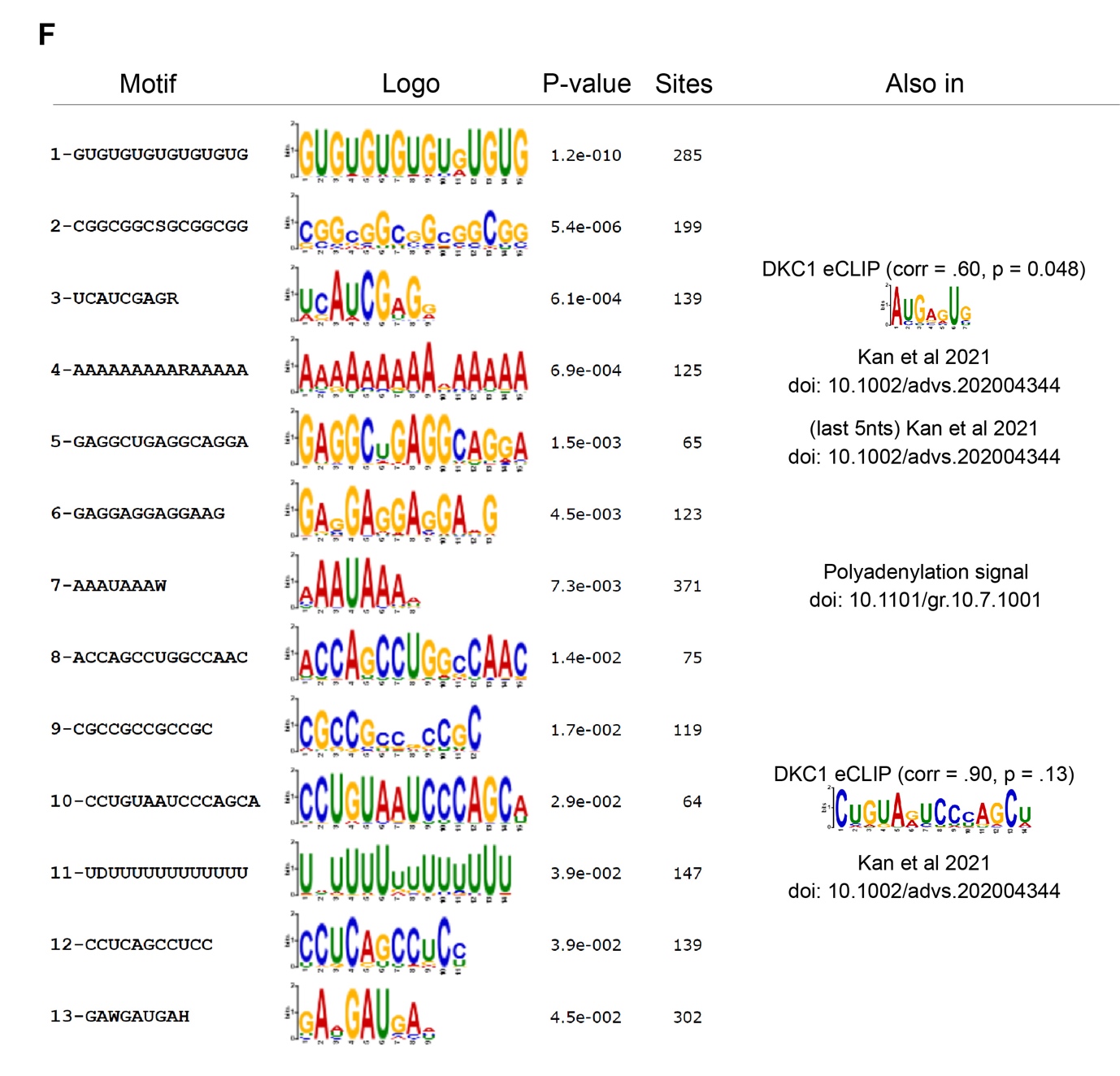


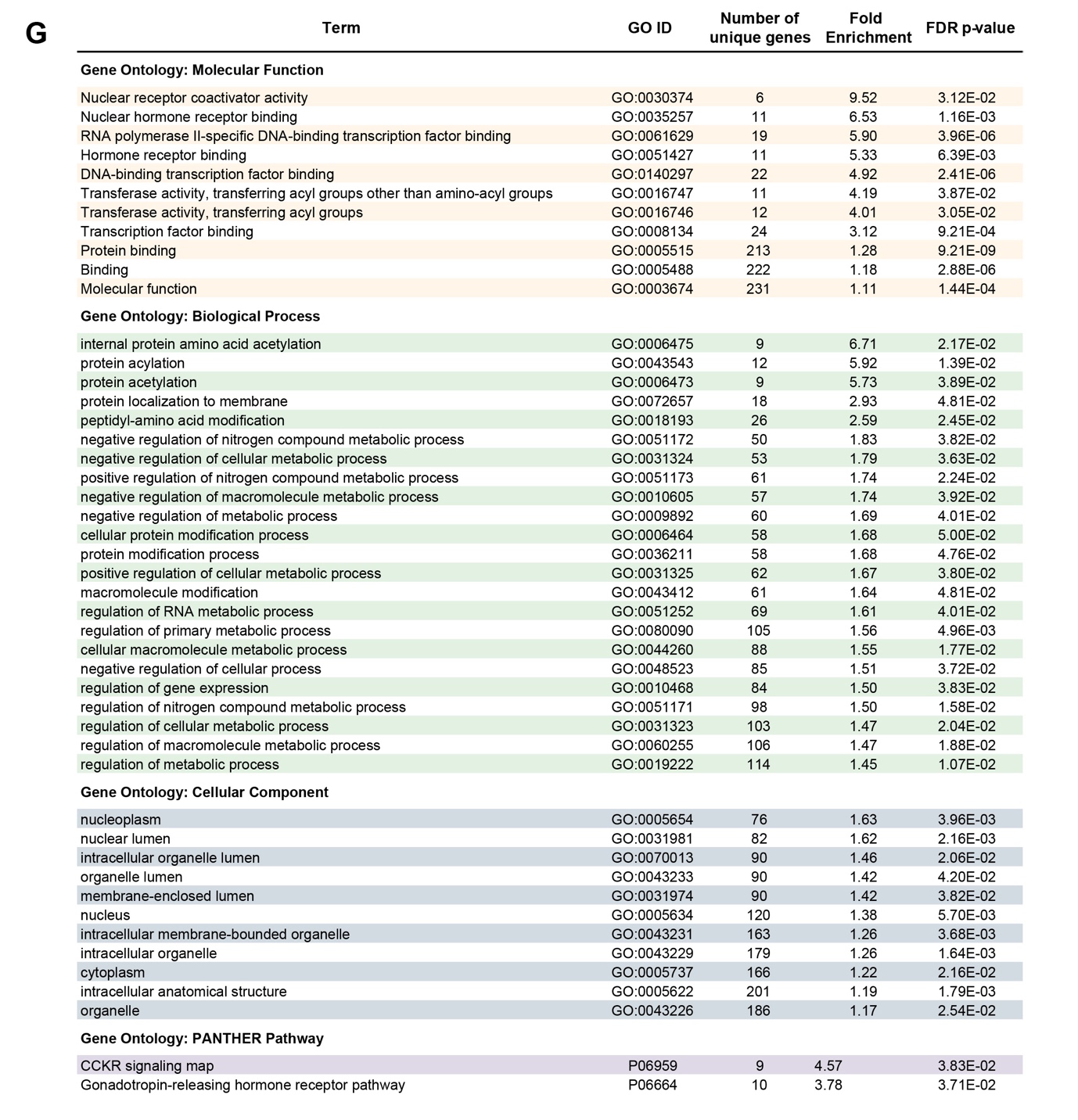


**Fig S4**, related to Figure 4. **Dyskerin binds to a complex RNA interactome in the cytoplasm and these genes are regulated after dyskerin partial depletion.**

**A** List of transcript biotype distribution and the number of genes identified by RIP-Seq analysis shown in Figure 4A.

**B** Sequencing of the 3’ EIF4A2-SNORA81 snoRT fragment (top) and the 3’ TAF1D-SNORA40 snoRT fragment (bottom) by 5’ RACE. The sequence of the 5’ portion of the transcripts are represented. The arrows indicate the two (for EIF4A2) o eight (for TAF1D) possible starting bases after the poly-(T) tail added during the 5’ RACE. On the right are represented the schemes of the two snoRTs and their relative 3’ snoRT fragments. The sequences of the two genes, focused on their relative snoRNA introns, are showed. Exons flanking intron-containing-snoRNA sequence are depicted as blue boxes or sequences, while the retained intron is depicted as a red box or sequence. The colored arrows represent the primers used for the 5’ RACE. In particular, for EIF4A2-SNORA81 snoRT the primers were: orange primer: “EIF4A2 snoRT SNORA81 RV”; green primer: “EIF4A2 snoRT SNORA81 nested1 RV”; purple primer: “EIF4A2 snoRT SNORA81 nested2 RV”: For TAF1D-SNORA40 snoRT the primers were: orange primer: “TAF1D snoRT 1 RV”; green primer: “TAF1D snoRT SNORA40 nested1 RV”; purple primer: “TAF1D snoRT SNORA40 nested1 RV”. The snoRNA sequences are underlined. The black arrow indicates the possible starting bases of the snoRT fragments.

**C** Absolute (ABS) distribution of log2 (fold-change) in the shDKC1 vs CTRL MCF7 cells RNA-seq dataset at the transcriptome and polysome levels. These tables show the distribution for genes identified as differentially expressed in RNA-seq dataset (red boxplots) and after a re-analysis limited only to the 701 genes identified as targets by the RIP-seq analysis (light blue boxplots), at the gene and transcript level (top and bottom row, respectively). Paired Student’s t tests were performed. *p < 0.05, **p < 0.01, ***p < 0.005, ****p < 0.001.

**D** Scheme of the over-representation analysis by one-sided binomial test of the transcripts that were immunoprecipitated by dyskerin and a curated list of known pseudouridylated transcripts.

**E** List of known pseudouridylated RNAs that are also found enriched in the dyskerin RIP-seq analysis with predicted H/ACA box snoRNAs obtained by snoGPS. For each transcript, the sequences of ten nucleotides around the pseudouridylated positions were used to obtain the predicted H/ACA box snoRNA and only snoRNA with a score more than 30 was indicated.

**F** Sequence motifs in the DKC1 cytoplasmic RNA immunoprecipitation*.* Figure shows the thirteen significant motifs (at p <= 0.05) found by analysis with STREME of the sequence of the 701 transcripts found to be bound by DKC1 in our cytoplasmic RNA immunoprecipitation experiment. Shown are the motif consensus, its weblogo, p-value and the number of sites composing the motif. Furthermore, similar motifs in other DKC1 immunoprecipitation experiments are also shown (Kan et al 2021 paper, and the ENCODE DKC1 eCLIP in HepG2 cells). Kan motifs were displayed in the related paper, while the eCLIP motifs were obtained by analyzing bound sites sequences with STREME, using the same parameters. Pearson correlation between the RIP and eCLIP motifs is also indicated).

**G** List of all statistically significant gene ontology terms broken down by molecular function, biological process, cellular component, and for the PANTHER pathways.


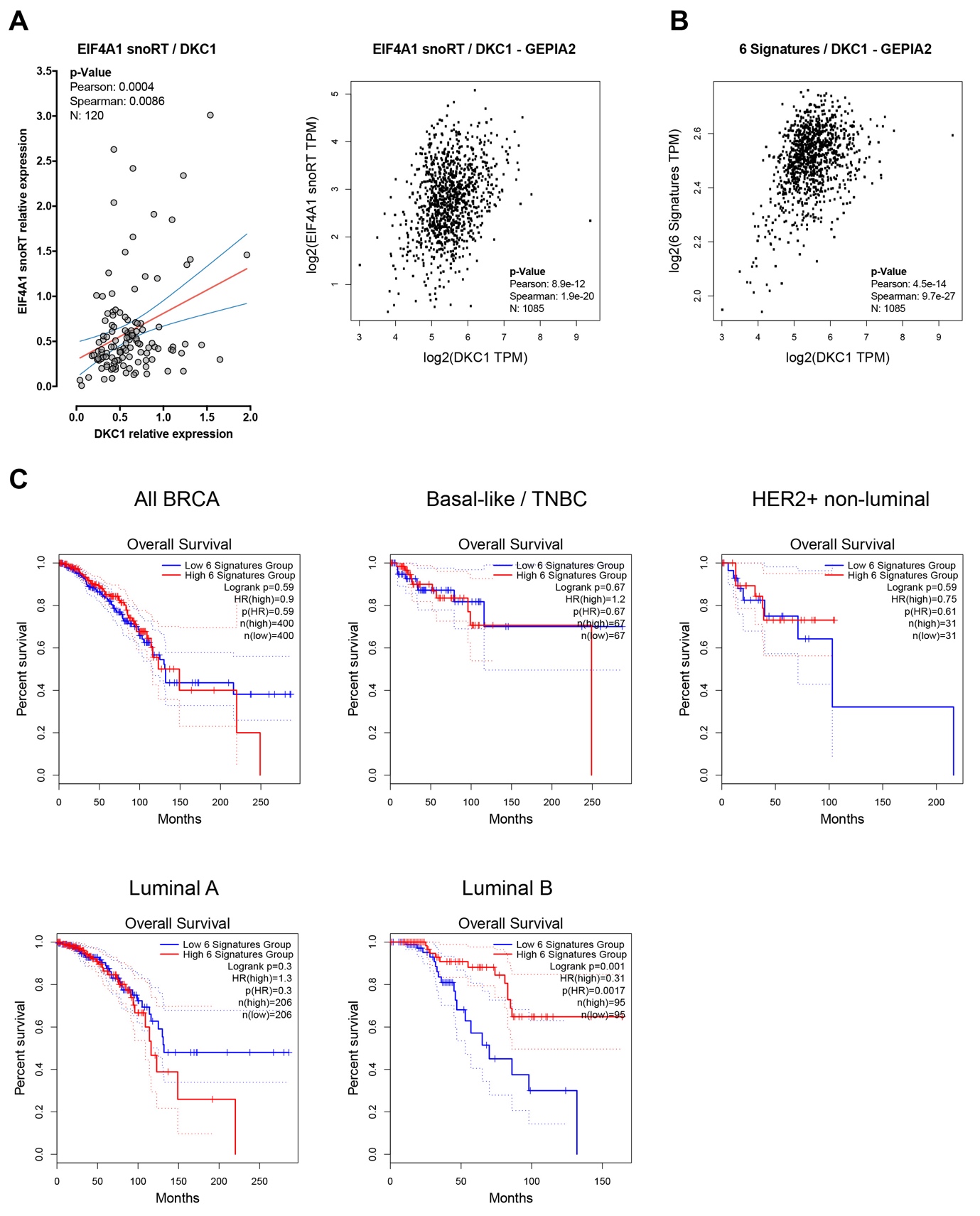


**Fig S5**, related to Figure 5.

**A** Left: Correlation between DKC1 and EIF4A1 snoRT relative expression in RNA extracted from 120 breast cancer tissue samples. Data obtained by RT-qPCR. The blue lines represent 95% C.I., while the red line is the best-fitting line. Pearson and Spearman correlation coefficient test was performed.

Right: Correlation between DKC1 and EIF4A1 snoRT relative. The analysis was performed using the TCGA breast invasive carcinomas dataset (1085 cases) by GEPIA2 algorithm. Pearson and Spearman correlation coefficient test was performed.

**B** Correlation between the relative expression of the signature of the 6 previously identified genes involved in “nuclear receptor coactivator activity” and “nuclear hormone receptor binding” molecular function and the expression of DKC1. The analysis was performed using the TCGA breast invasive carcinomas dataset (1085 cases) by GEPIA2 algorithm. Pearson and Spearman correlation coefficient test was performed.

**C** Kaplan-Meier survival curves of the signature of the 6 identified genes for breast cancer patients using the TCGA breast invasive carcinomas dataset (1085 cases) by GEPIA2 algorithm. Patients are divided between high and low 6 signature expression (separated by the median value). Dotted lines represent 95% C.I. Censored patients are indicated as the mark “I”. Log-rank (Mantel-Cox) test were performed.
