# Supplementary figures and images for: Human dyskerin binds to cytoplasmic H/ACA-box-containing transcripts affecting nuclear hormone receptor dependence
Source: Genome Biol. 2022 Aug 22;23:177. doi: 10.1186/s13059-022-02746-3 (PMC9394076; doi:10.1186/s13059-022-02746-3)

**Figure 1A**

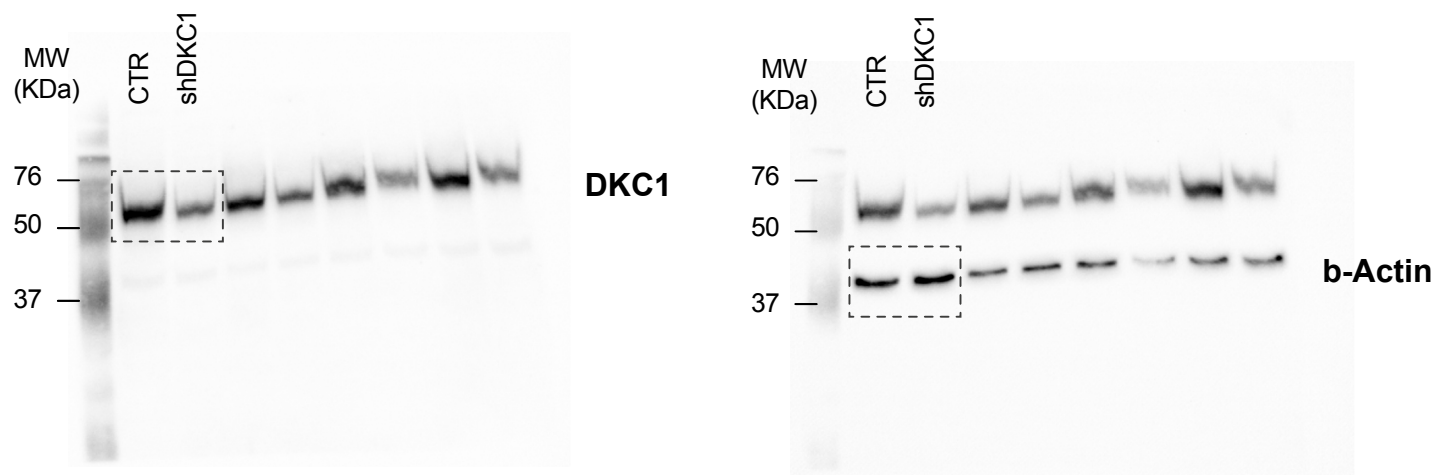

**Figure 2B**

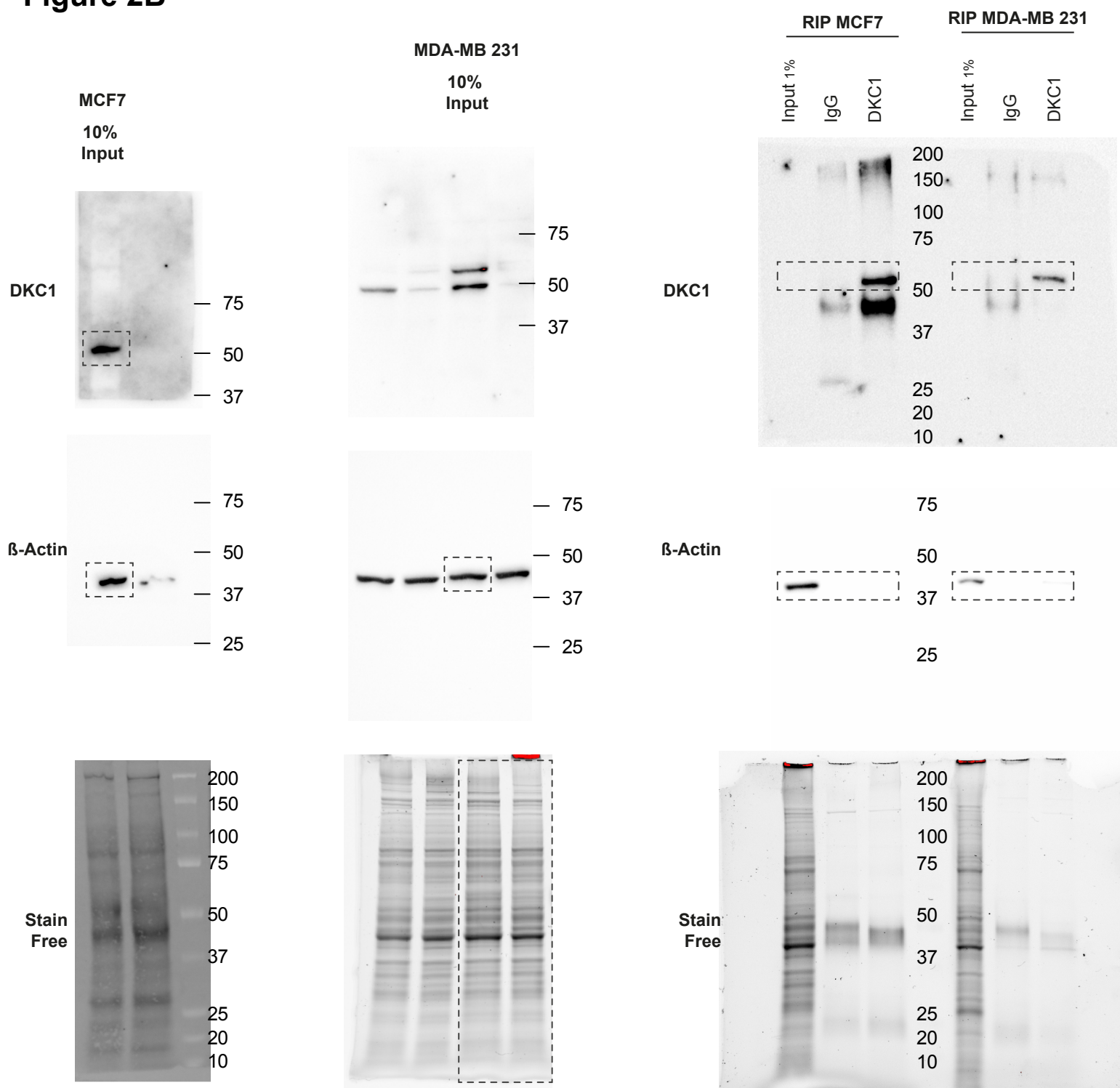

**Figure 2C**      10% Input

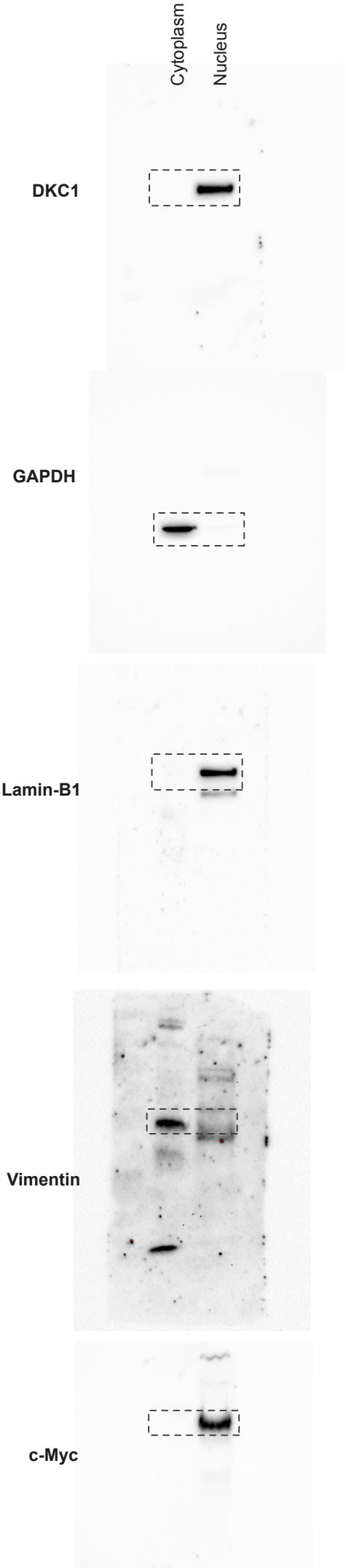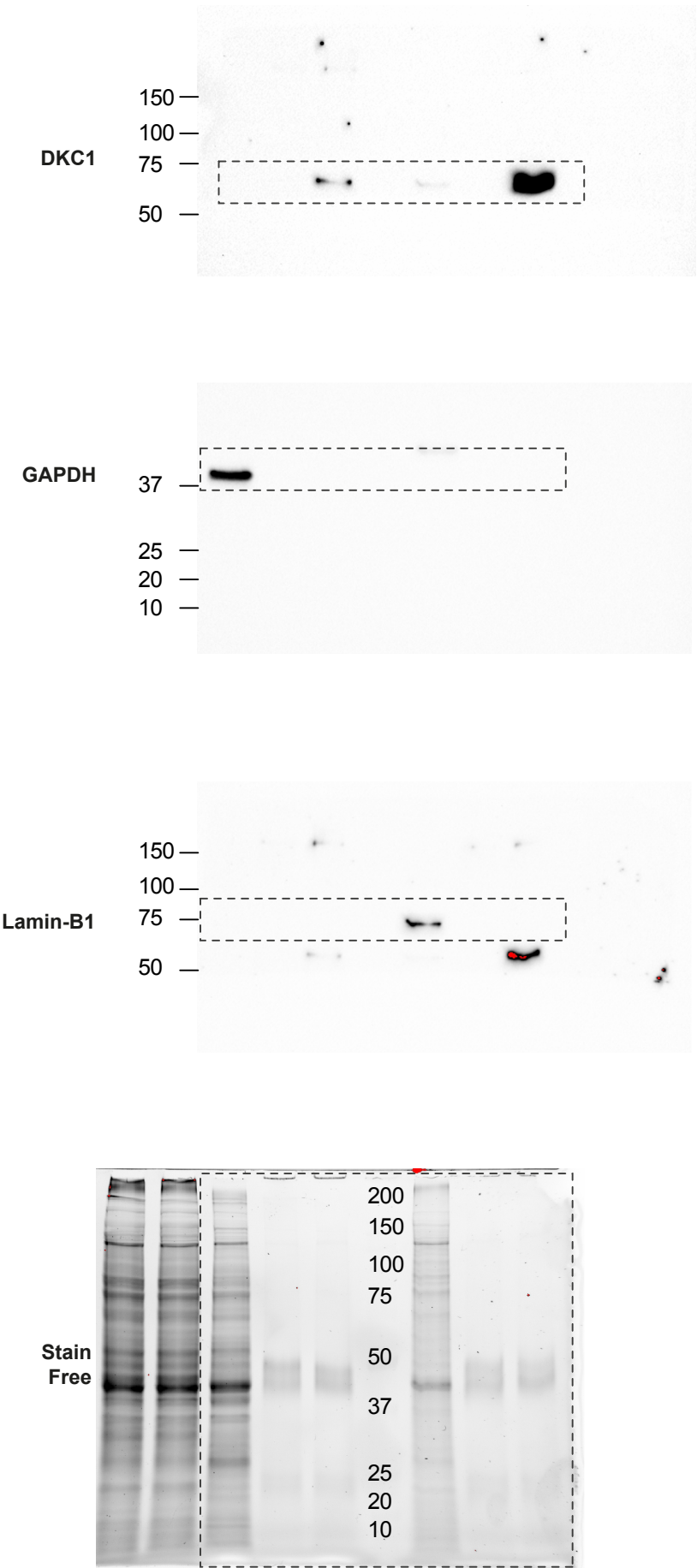

Figure 3E

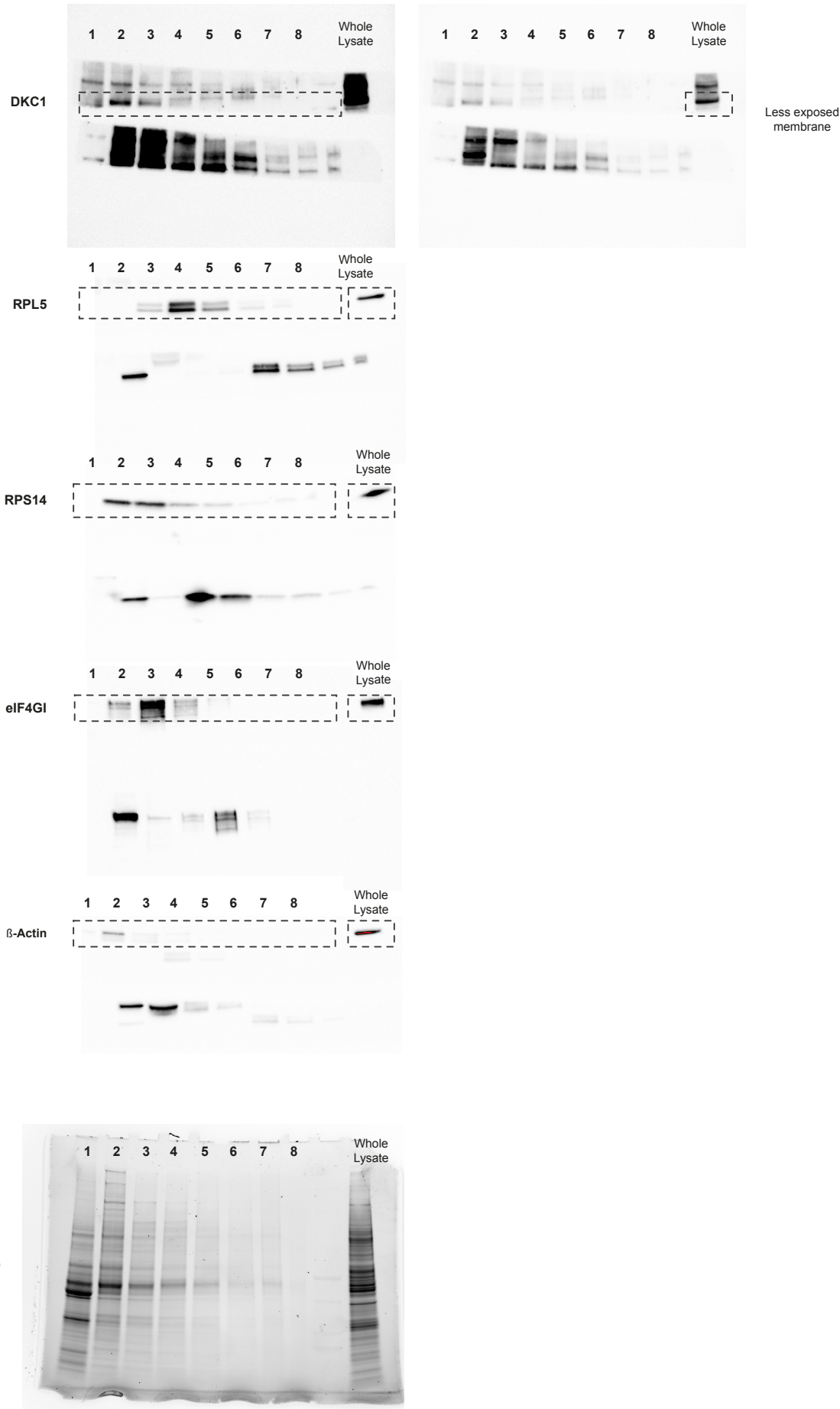

Figure 3F

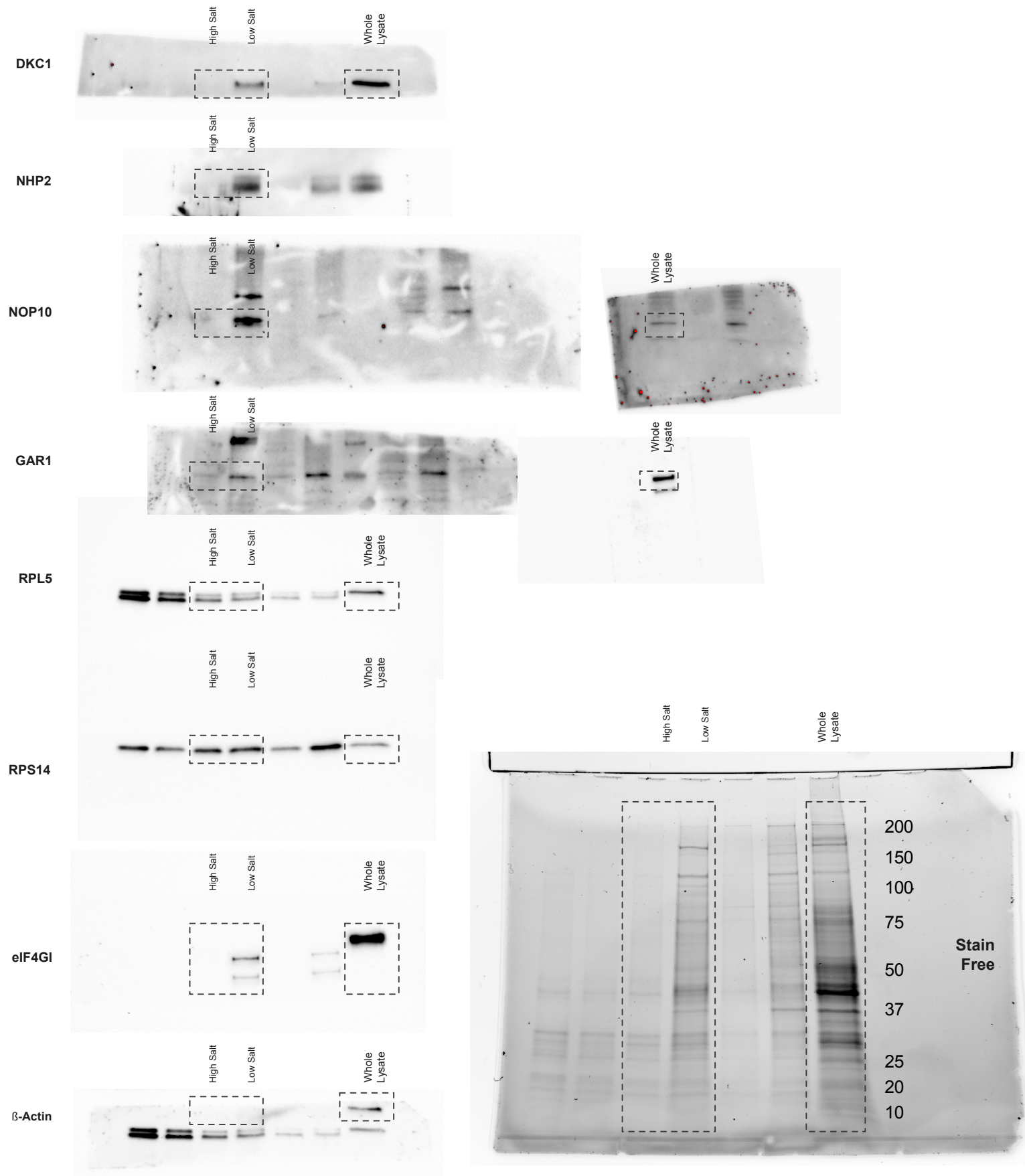

Figure 4C

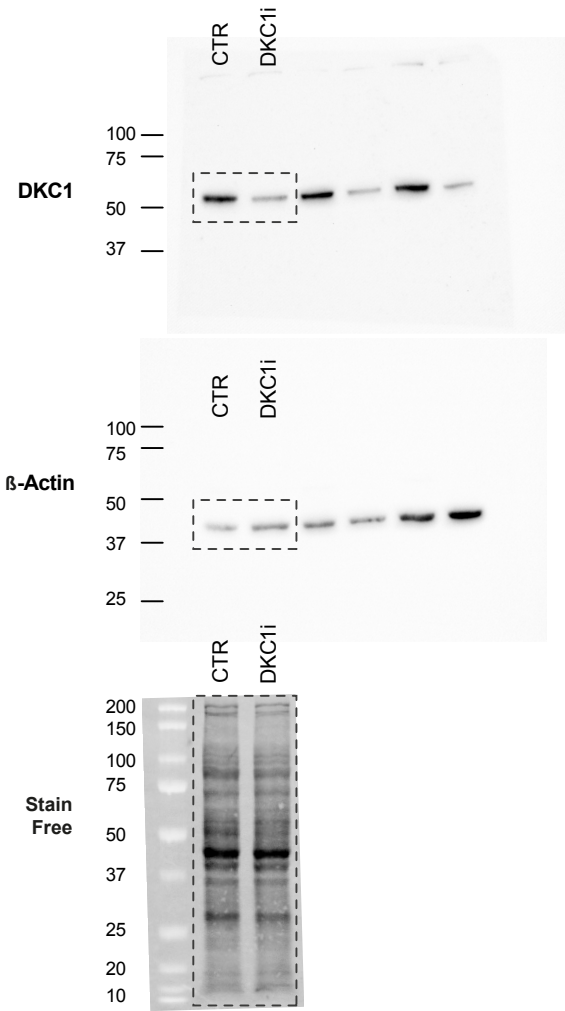

Supplement: Supplementary file 7 — Additional file 7. Uncropped Western Blot images. [file 13059_2022_2746_MOESM7_ESM.pdf]
