# Supplementary material for: Human dyskerin binds to cytoplasmic H/ACA-box-containing transcripts affecting nuclear hormone receptor dependence
Source: Genome Biol. 2022 Aug 22;23:177. doi: 10.1186/s13059-022-02746-3 (PMC9394076; doi:10.1186/s13059-022-02746-3)
Supplement: Supplementary file 8 — Additional file 8. Review history. [file 13059_2022_2746_MOESM8_ESM.docx]

Review History

**First round of review**

**Reviewer 1**

**Were you able to assess all statistics in the manuscript, including the appropriateness of statistical tests used?**

No.

**Comments to author:**

The manuscript by Zacchini et al., examines the effect of knocking out the snoRNA binding protein and pseudouridylase DKC1 on mRNA expression and association with the polysome. The authors identified specific mRNA isoforms that retain H/ACA snoRNA in their introns and associate with polysome in DKC1 dependent manner. These transcripts are truncated at the 5' end and bind to DKC1 in the cytoplasm. RNA co-immunoprecipitation assay indicates that DKC1 binds to a multitude of mRNAs that either contain intron embedded snoRNA, has potential snoRNA binding site or contain possible direct DKC1 binding motifs. Through gene ontology analysis the authors identified a subset for DKC1 bound mRNA coding for hormone receptors proteins and included experiments linking DKC1 to hormone response of breast cancer cells. The study includes several intriguing observations that could potentially be of high impact. However, in its current form the manuscript is difficult to read and the mechanisms by which DKC1 may affect gene expression, polysome association and cancer biology remain unclear. As presented, the manuscript is a collection of interesting DKC1 related phenomena that are not bound by a common mechanism or function. The link to hormone response and cancer biology applies only to a subset of DKC1 target while the function of the vast majority of DKC1 interaction events remain unclear. It is also not clear, if DKC1 regulates translation, splicing, RNA stability directly, via RNA modification or through multiple mechanisms. The origin and location of the binding events is not well described. Is the DCK1 / RNA binding initiated in the nucleus or occur de novo in the cytoplasm of free DKC1 retained in the cytoplasm or exported from the nucleus. Overall, the manuscript would benefit from streamlining and focusing on one central mechanism or group of substrates. For example, it might be useful to focus only on the RNA and mechanisms of action leading to DKC1 dependent modification of hormone response and cancer.

Specific Comments:

1. The origin and function of DKC1 dependent snoRT is not clear. Do these transcripts represent splicing anomaly, regulatory program or tools for increasing protein diversity?

2. Does DKC1 bind to snoRT in the nucleus or the cytoplasm?

3. Does DKC1 binding to snort precede spliceosome binding leading to splicing inhibition and the accumulation of unspliced RNA or does it bind to residual amount of unstable splice variants resulting from failed or inefficient splicing?

4. Does DKC1 one affects the association of snoRT with the polysome by physically blocking the association of the ribosomal subunits or indirectly alters translation by modifying the RNA? Indeed, it is not clear if DKC1 remains associated with the RNA during translation or not. It might be interesting to use FISH analysis to determine the location of DKC1 bound RNA in the cytoplasm and its relation to active ribosome.

5. The authors suggest that DKC1 may promote or inhibit association with the polyribosome, but they do not provide explanation of which RNA is promoted and which inhibited and why. Promotion and inhibition of translation require very different mechanism of action and it is curious to see how a single protein may lead to these two different effects.

6. The fate and merit of snoRT association with polyribosome are not clear. Are snoRT translated, associate non-productively with ribosome or block translation. Are the snoRT truncated peptides detected? Where do snoRT translation stop before, within the snoRNA sequence or after the snoRNA sequence? What is the location of the stop codon generated by the inclusion of the snoRNA in the transcript?

7. Are the cytoplasmic DKC1 / snoRNA, DKC1 / snoRT and DKC1 / mRNA complexes contain the rest of the canonical snoRNA binding proteins or each class of binding includes different protein partners?

8. The primer-pairs used for RT-qPCR of total and nuclear RNA can detect both pre-mRNA, full and truncated snoRTs. How do the authors differentiate between these different transcripts?

9. Transcripts that bind DKC1 in the cytoplasm should be classified based on the mechanism of binding (e.g., binding through intron embedded snoRNA in cis, binding through snoRNA guides in trans, snoRNA independent binding). Example of ach mode of binding should be verified experimentally.

10. The role of NMD and other ribonucleases in controlling the stability of each class of DKC1 interacting RNAs is not clearly described. The sensitivity of all DKC1 bound RNA to depletion of NMD factors and key cytoplasmic nuclear and cytoplasmic ribonucleases should be examined using published or in house genome-wide sequencing data.

11. Northern blot analyses of few examples of DKC1 interacting RNA will help clarify the identity and stability of the RNA isoforms affected by DKC1 (e.g., excised snoRNAs, EIF4A1-snoRT truncated, full length EIF4A1-snoRT and the mRNA).

12. What is the impact of DKC1 on the expression of the host genes carrying its targeted snoRNA? Do snoRTs regulate the translation of their host gene. It the increased association of snoRTs with polyribosome results in decreased association with normally spliced host mRNA? Western blot analysis of few examples would also be interesting in identifying the interplay between the snoRTs and host gene translation. For example, are the levels of EIF4A1 protein correlate or anticorrelate with the amount of total or polysome associated EIF4A1-snoRT.

13. What is the similarity and differences between the proposed snoRTs function and the previously described regulatory mechanism of Nop56/SNORD86?

14. In Figures S1E and 4C, the loading control is not constant across lanes and show possible DKC1 dependency and as such complicate the interpretation of the figure. A more stable loading control should be used. Otherwise, adding the value of the adjusted quantified bands after each gel might be helpful.

15. It is not clear why the authors use two different methods to deplete DKC1 using siRNA and shRNA). The use of the transient depletion using siRNA should be justified.

16. It is not clear how the authors discriminate between bonified cytoplasmic interaction and interactions occurring prior to cell lysis. Perhaps it is better to use in vivo cross-linking techniques to verify in vivo interactions.

17. It is not clear if the sequencing techniques use are capable of accurately and efficiently detect mature snoRNA. It is well known that most reverse transcriptases do not efficiently transcribe snoRNA and as such might bias the data towards truncated and intron embedded snoRNA, which are generally are less structured than their mature snoRNA counterpart. Northern blots or other reverse transcription techniques should be used to identify the real ratios of mature and intron bound snoRNAs

18. The Figure 3A is confusing as the two panels are hard to compare. It is difficult to ascertain whether "EIF4A1 snoRT is the most regulated transcript" from Figure 3A as claimed by the authors.

19. Figure S4C is confusing, why are the transcripts and gene analysis give opposite conclusions? In addition, it is not clear what the X-axis represents. It would be easier to understand the figure if only the absolute fold change is shown.

20. Line 191, it is not clear how the authors arrived at the frequency of known pseudouridylation sites in DKC1 bound RNA (i.e., where the number 356 comes from) Shouldn't the number include all known pseudouridylation sites (4128/ 87935)?

21. The authors identified 114 DKCS1 interacting transcripts containing pseudouridylation modifications. It would be interesting to investigate whether the modifications are occurring close to the binding site of DKC1. Do DKC1 biding sites correlate with the position of RNA modification. Is the binding and modification are linked or represent two separate events with different functional outcome?

22. The authors suggest the DKC1 may directly bind to RNA independent of the guide snoRNA. They have identified 13 significant motifs but have not specified the proportion and type of transcripts containing these motifs. Are these motifs present only in the absence of intron embedded snoRNA (snoRTs) and snoRNA binding (modification) site or they are randomly distributed? Are these motifs sufficient and necessary for the DKC1 binding? Do they affect polyribosome association or RNA stability?

23. The mechanism of DKC1 binding to genes involved in hormone response is not well described. What type of hormone response RNA bind to DKC1? Are they snoRTs, independent binders, mRNA, or modified mRNA? In all cases, snoRNA independent mRNA pseudouridylation of hormone response genes by DKC1 should be carefully validated if indeed it is the main mechanism of regulation for this subclass.

24. In Figure 1B, the polyribosome gradient shows very little polyribosome suggesting that translation is either inhibited or that most ribosome dissociate during extraction or preparation. In either case, it is unlikely that this type of gradients accurately represents the distribution of actively translated mRNA. Most cytoplasmic RNA should be engaged with polyribosome under normal condition, which does not appear to be the case in this gradient. The validity of the findings and suitability of the gradient should be confirmed by following the translation of few RNA using PCR or other targeted methods in a more standard polysome enriched gradient. It would be interesting to use mRNA with established translation pattern control. Comparison between the 40S, 80S and polyribosome bound mRNA population may also give us a more accurate indication of the mechanism by which DKC1 alter translation. Does DKC1 affect translation initiation or elongation?

**Reviewer 2**

**Were you able to assess all statistics in the manuscript, including the appropriateness of statistical tests used?**

Yes

**Comments to author:**

This is a very interesting study that unveils a new cytoplasmatic function for the pseudouridine synthase and RNA binding protein Dyskerin, in controlling pos-transcriptional gene expression. The authors find that Dyskerin binds selected mRNA isoforms characterized by the retention of H/ACA box snoRNA-containing introns that they named snoRTs. These snoRTs can be processed in the cytoplasm in in the form of shorter 3' snoRT fragments. The authors then focused on the characterization of one specific snoRTs, the EIF4A1 snoRT, which is associated with translating ribosomes. It would be important to assess whether these mRNA isoforms are being translated and what the effect is of downregulating EIF4A1 snoRT on cellular homeostasis.

The author further performed RNA immunoprecipitation (RIP) to characterize the Dyskerin RNA interactome in the cytoplasm. After an extensive analysis, the RIP data suggests that DKC1 may bind different transcripts either through the H/ACA snoRNA binding sequences or through additional recognition sequences. Interestingly, a subgroup of Dyskerin-bound mRNAs are important for nuclear hormone receptor ligand response. Strikingly, the authors showed that breast cancer cells harboring a decrease in Dyskerin expression can grow without the presence of hormone. These results are highly significant and suggest that human breast cancer cells can overtime be under a selection pressure to lose Dyskerin in order escape from hormone dependency and became more aggressive. This important and exciting manuscript should be published in Genome Biology. I only have a few comments that may strengthen the conclusions of this work.

Specific comments:

1. Can the authors explore the effect of DKC1 levels on additional mRNA isoforms such as CASP7, THOP1, RPS11?

2. The authors should assess whether EIF4A1 full length snoRT, 3' EIF4A1 snoRT and coding canonical EIF4A1 mRNAs found associated on the polysomal fractions are being translated.

3. Does Dyskerin sediment with 3' EIF4A1 snoRT on the polysome?

4. It would be informative to assess the specific effects of silencing EIF4A1 snoRT on cellular homeostasis.

5. Are all the components of the pseudouridylation complex (DKC1, NOP10, NHP2, GAR1) associated with cytoplasmic ribosomes? And if this is the case can the authors assess if this association is due to the presence of snoRTs?

**Authors Response**

**Point-by-point responses to the reviewers’ comments:**

**Reviewer #1**

1. The origin and function of DKC1 dependent snoRT is not clear. Do these transcripts represent splicing anomaly, regulatory program or tools for increasing protein diversity?

*Most of the snoRTs are non-coding transcripts. Specifically, only 3 out of 50 (56 in the present version of the manuscript – see point 9 below) snoRTs we identified in our model cell line by RIP-seq analysis are reported to be coding. Therefore, we assume they do not play a significant role in increasing protein diversity. On the other hand, previous reports and the analysis of public datasets indicate that snoRTs are abundantly represented in different cell types and tissues, also from non-pathological sources. This suggests that they should not be considered as the result of an infrequent splicing anomaly (Yin et. al. PMID: 22959273; Zhang et. al. PMID: 24734784; Xing et. al. PMID: 28475895; Lykke-Andersen et. al. PMID: 25403180; review in Wu et. al. PMID: 28629949 and Wong et. al. PMID: 26612485). On the other hand, our results (Figure 4) indicate that they can be involved in a regulatory program. These considerations have been added to the Discussion section of the manuscript (line 444-448).*

2. Does DKC1 bind to snoRT in the nucleus or the cytoplasm?

*This is a relevant question and we thank the Reviewer for pointing this out. Our results indicate that snoRTs are found associated with dyskerin also in the nucleus, suggesting that they are bound in the nucleus and then exported in the cytoplasm where they are accumulated. In addition, the binding of H/ACA box factors to snoRNA sequences require a complex biogenesis, with the intervention of processing factors such as NAF1 and SHQ1 in the nucleus. It is therefore unlikely that the binding can occur in the cytoplasm. Consistently, in our DKC1-IP experiments we could pull-down all the studied snoRTs both from nuclear and cytoplasmic fractions. Specifically, our data on EIF4A1-snoRT show that, in the cytoplasm, dyskerin is mainly associated to 3’snoRT while very little is associated to the full length snoRT transcript. We now added the evaluation of full length EIF4E1 snoRT after DKC1-IP from nuclear and cytoplasmic fractions. This analysis showed that while the full-length (unprocessed) transcript is weakly associated with dyskerin in the cytoplasm it is instead enriched in the nuclear fractions. These results are consistent with the binding of dyskerin to the full-length snoRT in the nucleus, its export in the cytoplasm and its processing to 3’snoRT fragment. These IP data have been added to the manuscript (Figure 3C – right panel and results section lines 186-188).*

3. Does DKC1 binding to snort precede spliceosome binding leading to splicing inhibition and the accumulation of unspliced RNA or does it bind to residual amount of unstable splice variants resulting from failed or inefficient splicing?

*To answer this point and characterize the contribution of dyskerin levels on the retention of snoRNA-containing introns we assessed the levels of total and newly produced full-length EIF4A1 snoRT after DKC1 modulation. Our results show that both total and newly transcribed levels of full-length EIF4A1 snoRT transcript are not influenced by dyskerin, indicating that intron retention is not influenced by the pre-existing levels of DKC1. These results have been added to the results section (lines 188-193) and Figure S3C.*

4. Does DKC1 one affects the association of snoRT with the polysome by physically blocking the association of the ribosomal subunits or indirectly alters translation by modifying the RNA? Indeed, it is not clear if DKC1 remains associated with the RNA during translation or not. It might be interesting to use FISH analysis to determine the location of DKC1 bound RNA in the cytoplasm and its relation to active ribosome.

*We thank the Reviewer for pointing out this important issue. To answer it we added data on polysomal fractioning in control conditions and after administration of inhibitors of mRNA translation (as also requested by Reviewer n. 2). Obtained results show that EIF4A1 snoRT is translated and dyskerin is found associated to polysomes. However, the majority of processed 3’EIF4A1 fragments and dyskerin are not found associated to polysomes but co-sediments with the small ribosomal subunit. These results are consistent with the hypothesis that the dyskerin-bound full length EIF4A1 snoRT transcript is recruited to the polysomes and translated, thus activating RNA decay mechanisms leading to the accumulation of the 3’EIF4A1 fragment outside polysomal fractions. These results are now reported in the results section (lines 201-221) and in Figure 3E and Figure S3F.*

5. The authors suggest that DKC1 may promote or inhibit association with the polyribosome, but they do not provide explanation of which RNA is promoted and which inhibited and why. Promotion and inhibition of translation require very different mechanism of action and it is curious to see how a single protein may lead to these two different effects.

*In the present study we initially identify a limited number of transcripts (i.e. CASP7, THOP1, RPS11) whose translation is affected in different direction by DKC1 depletion. We now add (also answering a point from Reviewer n. 2) the characterization of the effect of DKC1 depletion on the levels of these transcripts and of the encoded proteins (results section line 95 and Figure S1D). The complex remodulation that dyskerin exerts on mRNA translation was already observed previously (see for instance Rocchi et al. 2013, Nucleic Acid Research).*

*Coming down to snoRT, only the RPL32 transcript resulted to be favored in translation after DKC1 depletion. Interestingly it appears to be the only coding snoRT in the list we identified. This could be the reason why its recruitment to polysomes is favored by DKC1 depletion.*

6. The fate and merit of snoRT association with polyribosome are not clear. Are snoRT translated, associate non-productively with ribosome or block translation. Are the snoRT truncated peptides detected? Where do snoRT translation stop before, within the snoRNA sequence or after the snoRNA sequence? What is the location of the stop codon generated by the inclusion of the snoRNA in the transcript?

*As mentioned for point n. 4, we added data on EIF4A1 on polysomal fractions +/- puromycin showing snoRT association to polyribosomes and their translation. Again, we have data on EIF4A1 (and TAF1D and RPL32) demonstrating that snoRTs are actually recruited to the polysomes and translated. This observation is further corroborated by publicly available ribosome profiling data that we now add to the Results section (line 214 – Figure S3F).*

*The anti-EIF4A1 antibody we used binds to an epitope located in the initial polypeptide sequence (aa 4-23). However by Western blot analysis we were not able to identify any EIF4A1 truncated peptides.*

*Considering the canonical reading frame, the stop codon in snoRTs can be easily predicted and is generally located in the retained intron before the SNORA sequence (the location of the predicted stop codons is now indicated in the supplementary figure S3A showing the snoRT sequence).*

7. Are the cytoplasmic DKC1 / snoRNA, DKC1 / snoRT and DKC1 / mRNA complexes contain the rest of the canonical snoRNA binding proteins or each class of binding includes different protein partners?

*In the first version of the manuscript we provided RNA-IP data showing that EIF4A1 snoRT can be immunoprecipitated both by anti-dyskerin and anti-GAR1 antibodies. Considering that GAR1 is the outer component of the H/ACA complex and the last one to bind in H/ACA box RNP biogenesis, we may argue that snoRTs are bound also by the other core proteins. We now add RIP experiments performed with anti-NHP2 antibodies demonstrating that also this RNP core protein is associated with snoRTs (results section line 134 Figure S2G).*

8. The primer-pairs used for RT-qPCR of total and nuclear RNA can detect both pre-mRNA, full and truncated snoRTs. How do the authors differentiate between these different transcripts?

*The primer pairs used to detect the full-length EIF4A1 snoRT do not amplify the truncated snoRT since the forward primer falls in the exon at the 5’ of the retained intron that is removed in the truncated snoRT. In principle, these primers could amplify the unspliced pre-mRNA. To clarify this point we looked for the presence of other, non-snoRNA containing, EIF4A1 introns in the dyskerin IP. Obtained results indicate that the EIFA1 pre-mRNA does not associate with dyskerin (see results section lines 113-117 and Figure S2C).*

9. Transcripts that bind DKC1 in the cytoplasm should be classified based on the mechanism of binding (e.g., binding through intron embedded snoRNA in cis, binding through snoRNA guides in trans, snoRNA independent binding). Example of ach mode of binding should be verified experimentally.

*To answer to this point through experimental data, we analyzed an independently available anti-dyskerin eCLIP dataset (ENCODE Data Portal) obtained on HepG2 cells, looking for the transcripts we found enriched in our RIP-Seq analysis.*

*In this regard, we re-analyzed the enriched transcripts after dyskerin RIP-Seq according to the most recent Genebank version, finding 56 snoRT transcripts (in place of the 50 previously identified). This number was changed in the results and in Figure 4.*

*The analysis of the eCLIP dataset showed that most of the snoRT (52/56) and snoRNA/scaRNA sequences (104/115) that we identified in our RIP-Seq display a coverage enrichment, thus indicating that they could be bound directly (in cis) by dyskerin. Also, 71 additional transcripts show a similar behavior but they do not retain any reported snoRNA sequence. A further analysis on these transcripts showed that 48/71 contain one of the consensus sequences described by Kan et al. or those identified in our study.*

*All the remaining transcripts (including the majority of the coding ones – 174/312) do not show any enriched coverage in the CLIP analyses, suggesting they might by bound by dyskerin indirectly (in trans). We added this analysis in the results section (lines 319-336) and a table in the supplementary material (Supplementary Table S5) indicating the distribution of transcripts directly bound by dyskerin among those found enriched in our RIP-Seq analysis.*

10. The role of NMD and other ribonucleases in controlling the stability of each class of DKC1 interacting RNAs is not clearly described. The sensitivity of all DKC1 bound RNA to depletion of NMD factors and key cytoplasmic nuclear and cytoplasmic ribonucleases should be examined using published or in house genome-wide sequencing data.

*Based on published data (SRA Accession ID SRP063462 as reported in the Materials and Methods section) we monitored the sensitivity to NMD of the transcripts identified in our RIP-Seq analysis. In this analysis, 22/701 transcripts were found to be sensitive to NMD. Surprisingly, 15 of these belong to the protein coding subtype, while the only sensitive snoRT is TAF1D. This susceptibility has already been reported in the literature (Lykke 2014). We added this information to the Table S5 mentioned in point 9.*

11. Northern blot analyses of few examples of DKC1 interacting RNA will help clarify the identity and stability of the RNA isoforms affected by DKC1 (e.g., excised snoRNAs, EIF4A1-snoRT truncated, full length EIF4A1-snoRT and the mRNA).

*In this regard we would first like to point out that, given the initial findings and the consequently derived experiments, in our study we never looked specifically at excised, mature snoRNAs but instead only at transcripts retaining the snoRNA introns. During the revision process, due to technical difficulties we were not able to obtain clear Northern blot results for EIF4A1 snoRTs. To answer the issue raised by the Reviewer independently of the technical approach, we added data (Figure 3C – middle panel and results section lines 175-183) obtained by performing a digital PCR on MCF7 cells after DKC1 silencing by siRNA to evaluate the regulation of EIF4A1-derived RNAs. As described, this approach involves the use of primers specifically designed to distinguish the different isoforms, completely excluding the mature snoRNA sequence from the amplification process (see Figure 3B for amplification strategy). In order to exclude any possible issues related to the reverse-transcription of highly structured RNAs (like many snoRNAs are), in these experiments we performed cDNA synthesis using the TGIRT reverse transcriptase, which is able to operate at 60°C with higher thermostability, processivity, and fidelity than retroviral reverse transcriptases, allowing full-length, end-to-end cDNA synthesis also from highly structured or heavily modified RNAs and RNAs containing GC-rich repeat expansions. Obtained results showed that 3'EIF4A1 snoRT fragment is the only affected by DKC1 silencing, demonstrating that the regulation of the EIF4A1 snoRT observed in the RNA-seq is actually to be ascribed to downregulation of this specific RNA fragment. Furthermore, we observed very similar results performing this reverse transcription either with oligo-dTs or with random primers (see Figure S3B right panel), further demonstrating that with this assay we are actually evaluating mRNA isoforms and their derived fragments. Finally, similar results, although with milder regulation, were observed using MCF7 cells stably depleted for DKC1 (MCF7 shDKC1), consistent with the milder effect expected on DKC1 levels (figure below, not added to the manuscript).*

12. What is the impact of DKC1 on the expression of the host genes carrying its targeted snoRNA? Do snoRTs regulate the translation of their host gene. It the increased association of snoRTs with polyribosome results in decreased association with normally spliced host mRNA? Western blot analysis of few examples would also be interesting in identifying the interplay between the snoRTs and host gene translation. For example, are the levels of EIF4A1 protein correlate or anticorrelate with the amount of total or polysome associated EIF4A1-snoRT.

*Figure S1E already reported the effect of DKC1 silencing on the EIF4A1 coding transcript. In addition, we measured the level of the EIF4A1 protein after DKC1 silencing (result section line 92 and Figure S1D). No consistent changes were observed in both cases. Concerning the interplay between the snoRT and the host gene, also answering to Reviewer 2, we specifically silenced the snoRT by siRNA targeting the intronic region and evaluated the effect on the host gene expression by WB. Results are reported in Supplementary Figure S3H and result section lines 221-235. These results are in line with our initial RNA-Seq analysis on control and DKC1 KD cells, where the coding isoform of EIF4A1 mRNA is not affected by DKC1 depletion and EIF4A1 snoRT downregulation.*

13. What is the similarity and differences between the proposed snoRTs function and the previously described regulatory mechanism of Nop56/SNORD86?

*We added a specific sentence on this important point in the discussion section (page 15 lines 354 -358).*

14. In Figures S1E and 4C, the loading control is not constant across lanes and show possible DKC1 dependency and as such complicate the interpretation of the figure. A more stable loading control should be used. Otherwise, adding the value of the adjusted quantified bands after each gel might be helpful.

*We added the stain-free loading control allowing to evaluate the level of total proteins in each lane from the same blots in figures S1E and 4C.*

15. It is not clear why the authors use two different methods to deplete DKC1 using siRNA and shRNA). The use of the transient depletion using siRNA should be justified.

*DKC1 depletion by shRNA was preferred for polysomal analysis (e.g. the initial RNA-Seq approach) for quantitative reasons and for xenograft experiments in mice to obtain a stable DKC1 downregulation during the tumor growth. siRNA approach was preferred in experiments reported in Figure 4 to elicit a strong and acute DKC1 downregulation and in Figure S1E to confirm that effect of DKC1 KD by an independent approach. For the same reason, in the revised version of the manuscript the siRNA approach was used in experiments reported in Figure 3C, middle panel.*

16. It is not clear how the authors discriminate between bonified cytoplasmic interaction and interactions occurring prior to cell lysis. Perhaps it is better to use in vivo cross-linking techniques to verify in vivo interactions.

*We thank the reviewer for pointing out this important issue. We believe that in the present version of the manuscript, considering the addition of the results obtained by eCLIP analysis (see point 9), we have an independent confirmation that dyskerin actually interacts with snoRTs in live cells. In light of this confirmation, we feel that our quantitative observations on native endogenous protein-RNA interaction can be valued as more reliable.*

17. It is not clear if the sequencing techniques use are capable of accurately and efficiently detect mature snoRNA. It is well known that most reverse transcriptases do not efficiently transcribe snoRNA and as such might bias the data towards truncated and intron embedded snoRNA, which are generally are less structured than their mature snoRNA counterpart. Northern blots or other reverse transcription techniques should be used to identify the real ratios of mature and intron bound snoRNAs

*In this regard we would like to point out that in our study we never looked specifically to excised, mature snoRNAs but instead only to transcript retaining the snoRNA introns. To answer to this issue we added new data (see Point 11) (Figure 3C – middle panel, Figure S3B), performing a reverse transcription using the TGIRT reverse transcriptase which is considered the best approach to obtain quantitative results independently by complex RNA secondary structures.*

18. The Figure 3A is confusing as the two panels are hard to compare. It is difficult to ascertain whether "EIF4A1 snoRT is the most regulated transcript" from Figure 3A as claimed by the authors.

*We apologize with the Reviewer since that text was not clear on this issue. Specifically, we don’t claim that EIF4A1 snoRT is the most regulated transcript on the bases of Figure 3A but instead based on the results of RNA-seq reported in Figure 1C. Figure 3A provides an indication of the abundance of the reported transcripts. We changed that text accordingly (Results section, 3rd sub-section, line 135)*

19. Figure S4C is confusing, why are the transcripts and gene analysis give opposite conclusions? In addition, it is not clear what the X-axis represents. It would be easier to understand the figure if only the absolute fold change is shown.

*We agree with the Reviewer that Figure S4C could be confusing. We therefore removed two panels leaving only absolute fold change as suggested. Also, the labels of the groups reported in the X axis have been more clearly detailed in the figure legend.*

*We believe that the opposite conclusion obtained looking either at gene or transcript based results are representative of the key observations of the study (i.e. in the RNA-seq analysis reported in Figure 1 we could not see any differences at the gene level but we could see changes when performing a transcript-based or an exon-based analysis).*

20. Line 191, it is not clear how the authors arrived at the frequency of known pseudouridylation sites in DKC1 bound RNA (i.e., where the number 356 comes from) Shouldn't the number include all known pseudouridylation sites (4128/ 87935)?

*The number originally shown (356) was incorrectly reported, and did not correspond to the frequency indicated, we apologize for this. As the reviewer pointed out the correct number for this frequency is 4128/87935 and has been corrected in the text.*

21. The authors identified 114 DKCS1 interacting transcripts containing pseudouridylation modifications. It would be interesting to investigate whether the modifications are occurring close to the binding site of DKC1. Do DKC1 biding sites correlate with the position of RNA modification. Is the binding and modification are linked or represent two separate events with different functional outcome?

*As reported in the manuscript in our analysis we considered only the nucleotides flanking known pseudouridylation sites (and therefore potential snoRNA binding sites). Only in 11/114 transcripts the modified sites overlap the dyskerin binding site obtained from eCLIP analysis (see point 9 above). More than half of these transcripts (7) are snoRTs, in which the pseudouridylation site falls within the retained SNORA sequence. We believe that it is difficult to ascribe the site-specific pseudouridylation with the direct binding of diskerin to these sequences. In any case, we added this information to Table S5.*

22. The authors suggest the DKC1 may directly bind to RNA independent of the guide snoRNA. They have identified 13 significant motifs but have not specified the proportion and type of transcripts containing these motifs. Are these motifs present only in the absence of intron embedded snoRNA (snoRTs) and snoRNA binding (modification) site or they are randomly distributed? Are these motifs sufficient and necessary for the DKC1 binding? Do they affect polyribosome association or RNA stability?

*The occurrence and the distribution of known and putative dyskerin binding motifs has been reported in the Table S5. In particular, the table shows which motif coincide with the binding site of dyskerin identified by the above cited eCLIP analysis. Of the 13 motifs, one in particular is heavily enriched (motif-1) with 48 transcripts that present it and are bound by dyskerin in the same sequence. Interestingly, neither of these is a snoRNA or a snoRT, supporting the idea that some RNAs are bound independently of the presence of a snoRNA sequence. Other motifs were identified much less frequently as being actually bound by dyskerin in the eCLIP dataset (between 1 and 14 transcripts per motif), with about 50% of these transcripts belonging to snoRT or snoRNA biotype. Finally, 5 motifs do not have any correspondence between the motif sequence and the binding to dyskerin. This supports the idea that while some of the motifs identified actually correspond to a real dyskerin binding sites, this is less or not true for some of the other identified motifs. These observations have been added to the results section (lines 330-333). Concerning the effect of RNA stability on polysome association, our RNA-Seq analysis identified only a short list of transcripts which are quantitatively regulated by dyskerin. However some of them (CASP7, RPS11, THOP1) were not present in the RIP and so are not expected to be bound directly by dyskerin.*

23. The mechanism of DKC1 binding to genes involved in hormone response is not well described. What type of hormone response RNA bind to DKC1? Are they snoRTs, independent binders, mRNA, or modified mRNA? In all cases, snoRNA independent mRNA pseudouridylation of hormone response genes by DKC1 should be carefully validated if indeed it is the main mechanism of regulation for this subclass.

*All the 13 transcripts belonging to the “nuclear receptor coactivator activity” and “nuclear hormone receptor binding functions” are coding mRNAs (as indicated in the text the Gene Ontology analysis was performed only on the genes for which coding transcripts were identified in the RIP-seq analysis). None of them is a snoRNA-retaining transcript. Interestingly, 3 out of these 13 transcripts are known to be pseudouridylated (23,1%). The analysis of the sequences flanking these 3 modification sites identified 3 putative guide snoRNA/snoRT that turned out to be present in the RIP-seq analysis. This suggests that the modification of these 3 uridines could be RNA dependent.*

*This information has been added to the results section (lines 348-352).*

24. In Figure 1B, the polyribosome gradient shows very little polyribosome suggesting that translation is either inhibited or that most ribosome dissociate during extraction or preparation. In either case, it is unlikely that this type of gradients accurately represents the distribution of actively translated mRNA. Most cytoplasmic RNA should be engaged with polyribosome under normal condition, which does not appear to be the case in this gradient. The validity of the findings and suitability of the gradient should be confirmed by following the translation of few RNA using PCR or other targeted methods in a more standard polysome enriched gradient. It would be interesting to use mRNA with established translation pattern control. Comparison between the 40S, 80S and polyribosome bound mRNA population may also give us a more accurate indication of the mechanism by which DKC1 alter translation. Does DKC1 affect translation initiation or elongation?

*The shape of the polysomal profiles obtained in control and shDKC1 cells is typical for MCF-7 cells as reported by other authors in previous studies (e.g. Modelska et al. PMID: 25611378, Figure 4, Gandin at al. PMID: 24893926, Figure 1, D’Agostino et al. PMID 26553968, Figure 5). In this cell line the pre-polysomal peaks are generally higher than those of polysomes. As requested (also by Reviewer #2), we added the results obtained from additional polysomal fractions for some of our snoRTs and other coding transcripts (obtained on the MCF-7 parental cell line on different gradient ranges Figure 3E, lines 201-221) and the data on the effect of puromycin treatment on the translation of these mRNAs indicating that they are normally highly translated in standard conditions.*

**Reviewer #2:**

1. Can the authors explore the effect of DKC1 levels on additional mRNA isoforms such as CASP7, THOP1, RPS11?

*These are the significant transcripts deriving from the initial RNA-seq analysis. We added these data on isoform specific mRNA and protein levels (also answering a point from Reviewer n. 1). As already reported in Figure S1D, the expression of the transcripts is regulated by the partial depletion of DKC1. We have also added the western blots of the corresponding proteins (that however are not exclusively produced from the identified isoforms), showing that there are no appreciable changes in protein levels (see Figure S1D and results section line 92).*

2. The authors should assess whether EIF4A1 full length snoRT, 3' EIF4A1 snoRT and coding canonical EIF4A1 mRNAs found associated on the polysomal fractions are being translated.

*To answer this question, we added data on polysomal fractioning in control conditions and after administration of inhibitors of mRNA translation (as also requested by Reviewer n. 1). Obtained results show that EIF4A1 snoRT is translated and, consistently, dyskerin is found associated to polysomes. However, the majority of processed 3’EIF4A1 fragment and dyskerin are not found associated to polysomes but co-sediments with the small ribosomal subunit. A further confirmation that these transcripts are translated was obtained by analyzing public ribosome profiling data where it is evident that the SNORA67 intronic portion of the EIF4A1 transcript is bound by the ribosomes. These results are consistent with the hypothesis that the dyskerin-bound full length EIF4A1 transcript is recruited to polysomes and translated, activating RNA decay mechanisms leading to the accumulation of the 3’EIF4A1 fragment outside polysomal fractions. These results are now reported in the results section (lines 201-221) and in Figure 3E and Figure S3E-F.*

3. Does Dyskerin sediment with 3' EIF4A1 snoRT on the polysome?

*As reported for point 2, we observed that the majority of 3’ eIF4A1 fragments is found outside the polyribosomes and co-sediments with the 40S subunit (see results as indicated for Point 2 above).*

4. It would be informative to assess the specific effects of silencing EIF4A1 snoRT on cellular homeostasis.

*As also requested by Reviewer 1 we specifically depleted EIF4A1 snoRT using siRNA targeting the intronic retained sequence. While the silencing has no significant effect on the coding EIF4A1 mRNA isoform, the encoded protein and the total protein synthesis levels assayed by puromycin incorporation resulted to be reduced by EIF4A1 snoRT depletion. This was also associated with a slight negative effect on cellular growth of the silenced cells. These observations have been added to the results section (lines 221-235 and Figure S3H).*

5. Are all the components of the pseudouridylation complex (DKC1, NOP10, NHP2, GAR1) associated with cytoplasmic ribosomes? And if this is the case can the authors assess if this association is due to the presence of snoRTs?

*To answer to this point, we isolated ribosomes in low- and high-stringency purification conditions, demonstrating that all the pseudouridylation complex core proteins are associated to cytoplasmic ribosomes, paralleling what happens for other ribosome-interacting factors (e.g. eIF4GI - Figure 3F, Figure S3G). It appears difficult to specifically assess the role of snoRTs in this association since we don’t have a specific way to downregulate all the snoRTs. In this regard, however we observed that the inhibition of mRNA translation by puromycin treatment leads to the shift of both snoRTs and dyskerin from polysomal to pre-polysomal fractions (lines 212-215 and Figure S3E).*

**Second round of review**

**Reviewer 1**

The revised manuscript is much improved and most of my comments were adequately addressed. Some remaining issues may required further study but should not take from the importance of the study in its current form.

**Reviewer 2**

The authors addressed all my comments, and this manuscript is ready to be published. This superb work makes a significant advance to our understanding of gene expression at the post-transcriptional level and its implication in human disease.
